# From coherent shocklets to giant collective incoherent shock waves in nonlocal turbulent flows

**DOI:** 10.1038/ncomms9131

**Published:** 2015-09-08

**Authors:** G. Xu, D. Vocke, D. Faccio, J. Garnier, T. Roger, S. Trillo, A. Picozzi

**Affiliations:** 1 Laboratoire Interdisciplinaire Carnot de Bourgogne (ICB), UMR 6303 CNRS—Université Bourgogne Franche-Comté, F-21078 Dijon, France; 2 School of Engineering and Physical Sciences, SUPA, Heriot-Watt University, Edinburgh EH14 4AS, UK; 3 Laboratoire de Probabilités et Modèles Aléatoires, University Paris Diderot, 75205 Paris, 13, France; 4 Ecole Normale Supérieure, Department of Mathematics, CNRS, 45 rue d'Ulm, 75005 Paris, France; 5 Department of Engineering, University of Ferrara, Via Saragat 1, 44122 Ferrara, Italy

## Abstract

Understanding turbulent flows arising from random dispersive waves that interact strongly through nonlinearities is a challenging issue in physics. Here we report the observation of a characteristic transition: strengthening the nonlocal character of the nonlinear response drives the system from a fully turbulent regime, featuring a sea of coherent small-scale dispersive shock waves (shocklets) towards the unexpected emergence of a giant collective incoherent shock wave. The front of such global incoherent shock carries most of the stochastic fluctuations and is responsible for a peculiar folding of the local spectrum. Nonlinear optics experiments performed in a solution of graphene nano-flakes clearly highlight this remarkable transition. Our observations shed new light on the role of long-range interactions in strongly nonlinear wave systems operating far from thermodynamic equilibrium, which reveals analogies with, for example, gravitational systems, and establishes a new scenario that can be common to many turbulent flows in photonic quantum fluids, hydrodynamics and Bose–Einstein condensates.

## Abstract (web summary)

Understanding turbulent flows arising from random dispersive waves that interact through nonlinearities is a challenging issue in physics. Here, the authors model and observe experimentally in a nonlinear optics set-up the transition between a sea of small-scale shocklets and a giant collective shock wave.

## Graphical abstract

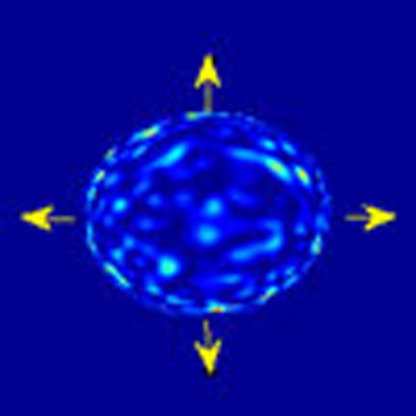

The statistical description of nonequilibrium behaviour of random dispersive waves is well developed in the weak nonlinear limit, for which wave turbulence theory provides a powerful tool to interpret observations arising in contexts as different as ocean waves, quantum fluids, plasmas and nonlinear optics to name a few^1^,^2^,^3^,^4^,^5^,^6^,^7^. However, when nonlinearities are strong, such an approach breaks down and no general theory exists. Therefore, novel scenarios that can be predicted starting from universal models and observed in real systems are of paramount importance. In this context, we explore how shock waves (at variance with other coherent structures such as vortices, quasi-solitons, collapsing wavepackets or rogue waves^1^,^2^,^3^,^6^,^7^,^8^,^9^,^10^,^11^,^12^,^13^,^14^,^15^,^16^) affect the turbulent flow of a conservative system of random optical waves (that is, a photon fluid) interacting through defocusing (or repulsive) nonlinearities. As a distinguishing feature, we consider the nonlocal character of the nonlinearity, which is indeed common to a large variety of nonlinear wave systems^17^,^18^,^19^,^20^,^21^,^22^,^23^,^24^,^25^,^26^,^27^,^28^,^29^,^30^,^31^,^32^. In such defocusing media, the generic signature of the strongly nonlinear regime is the formation of expanding undulatory structures known as dispersive shock waves (DSWs), which originate from the dispersion (diffraction) acting over steep fronts^33^ developing via the nonlinearity. These fascinating DSW structures^34^ have been observed from the small scales as in optics^24^,^35^,^36^,^37^,^38^ or in Bose–Einstein condensates^39^,^40^,^41^,^42^,^43^ to intermediate hydrodynamic scales^44^,^45^,^46^, or even long astrophysical spatial scales^47^.

In this paper, we report a remarkable transition of the turbulent flow that occurs in our photon fluid system when the characteristic range of nonlocality is increased. In the quasi-local regime, the field evolution is ruled by the stochastic formation of small-scale DSW structures that we naturally denote as dispersive ‘shocklets' (the term shocklet was introduced to designate sporadic steep fronts reported in high-speed compressible turbulence^48^ and as flank formations associated with planetary-scale shocks in space^49^). In this regime, wave breaking occurs at random positions in the turbulent field, predominantly around high-amplitude fluctuations, leading to a gas of coherent dispersive shocklets in the midst of turbulent fluctuations. In marked contrast with such regime, a remarkable self-organized regime emerges when the nonlinearity becomes highly nonlocal (long-range interaction). In this case, the turbulent flow follows an unexpected global collective behaviour that manifests itself in the formation of a giant shock singularity that emerges from the fluctuating field as a whole. Such a phenomenon is characterized by a strong non-homogeneous redistribution of the spatial fluctuations, whose description is provided in terms of a hydrodynamic-like model derived from singular solutions of a long-range Vlasov equation (LRVE). Our analysis reveals that (i) in marked contrast with coherent DSWs in conservative (Hamiltonian) systems, the regularization of the global incoherent shock does not require the formation of a regular oscillating DSW structure; and (ii) the self-organization of the turbulent wave ensemble into such a global shock structure is intimately related to the long-range character of the interaction, thus revealing interesting links with gravitational-like systems^50^,^51^. We emphasize that this regime is fundamentally different from other forms of turbulence that are dominated by inverse or direct cascades^1^,^2^,^6^,^7^,^8^,^12^, vortex dynamics^13^, acoustic turbulence^52^,^53^ or from the incoherent undulatory shocks that manifest solely in frequency domain in the weak Langmuir turbulent regime^54^. We directly observe the emergence of the giant collective incoherent shock phenomenon in an optical experiment that involves a graphene-based thermal medium, which allows for a tunable degree of nonlocality in the system.

## Results

### Nonlinear Schrödinger model

A nonlocal nonlinearity is found in several systems (dipolar Bose–Einstein condensates^26^, roton excitations in superfluids^28^, atomic vapours^23^, nematic liquid crystals^12^,^18^, thermal media^24^, glasses^19^,^20^,^21^,^25^, plasmas^27^ or plasmonic metamaterials^32^) and can be modelled by the following universal two-dimensional (2D) nonlinear Schrödinger equation (NLSE):


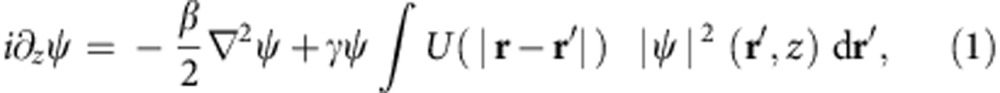


where the dynamics occurs in the transverse plane **r**=(*x*, *y*) and *z* plays the role of time. The parameters 
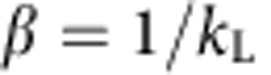
 and 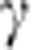 refer to the linear (dispersive) and nonlinear coefficients, *k*_L_ being the laser wave number. We denote by *σ* the spatial extension of the nonlocal response function *U*(*r*), that is, the range of nonlocal interaction. Equation (1) conserves two important quantities during the propagation, the ‘power' 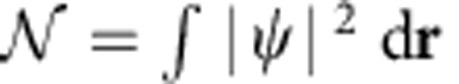, and the ‘energy' (Hamiltonian) 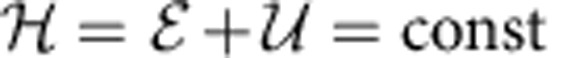, where 
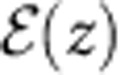
 and 
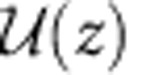
 denote the evolutions of the linear and nonlinear energy contributions (see Methods section). The dynamics is ruled by the comparison of 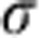 with the coherence length of the field 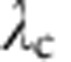, and the ‘healing length' 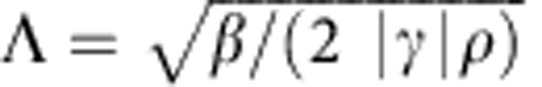, where 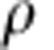 is the intensity. The healing length denotes the typical length scale for which linear and nonlinear effects are of the same order, for example, the typical size of a soliton or a vortex. In the following, we focus the presentation on the more interesting 2D defocusing regime, 
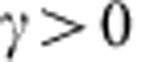
, while the analysis can easily be transposed to the focusing regime or to one spatial dimension (Supplementary Note 3).

### Local versus nonlocal regimes

**Figure 1:**
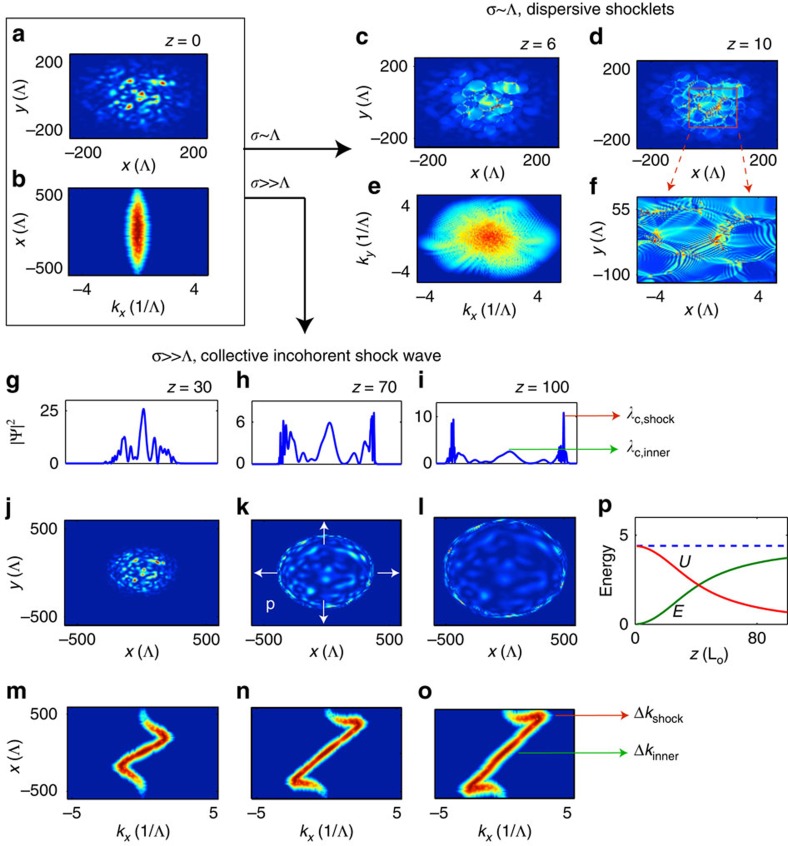
Dispersive shocklets versus giant incoherent shock. (**a**,**b**) Numerical simulations of the NLSE (1) starting from the incoherent wave 
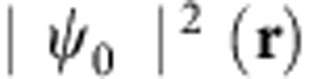
 (**a**) in the strong nonlinear regime, 
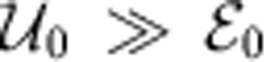
; (**b**) the spectrogram corresponding to this initial condition. (**c**–**f**) In the quasi-local regime 
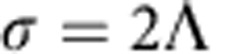
, the incoherent wave intensity develops a sea of dispersive shocklets (**c**,**d**), as evidenced by the formation of several DSWs (see zoom (**f**) of the red box in **d**, and the spectral filamentation in **e**). (**g**–**o**) Starting from the same initial condition (**a**), in the highly nonlocal regime 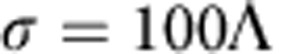, the random wave as a whole develops a giant collective incoherent shock (**j**–**l**); the corresponding intensity lineouts *y*=0 (**g**–**i**) indicate an annular collapse-like behaviour. (**m**–**o**) Corresponding spectrogram evolutions of the incoherent shock: the *Z*-shaped distortion reveals a dramatic coherence degradation on the annular boundaries of the beam (the correlation length decreases at the shock front, 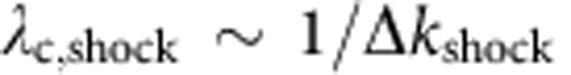), while a significant coherence enhancement occurs in the internal region of the beam (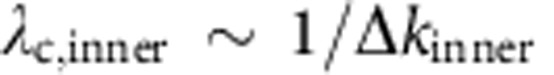 increases). (**p**) Evolutions during the propagation of the linear (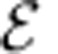, green) and nonlinear (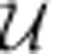, red) contributions to the total energy (constant Hamiltonian, 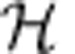, dashed blue): The incoherent shock develops in the strongly nonlinear regime 
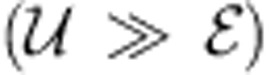
. The propagation length in the sample, *z*, is in units of the nonlinear length, 
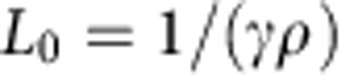
.

The formation of shock waves is known to require a strong nonlinear interaction^24^,^33^,^34^,^35^,^36^,^37^,^38^,^39^,^40^,^41^,^42^,^43^,^44^,^45^, that is, the initial random wave, 
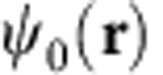
, is such that 
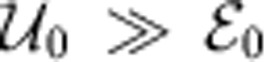
, where 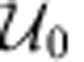 and 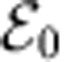 refer to the initial values of the energies at *z*=0 (Fig. 1a). Note that this condition is analogous to 
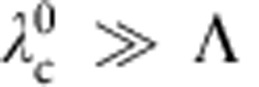
, 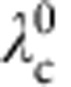 being the initial coherence length of the random wave, 
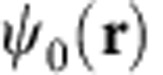
. Considering this strong nonlinear regime, we now contrast the case of a quasi-local (short-range) interaction, 
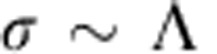
, with a highly nonlocal (long-range) interaction, 
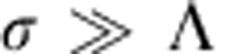
. For 
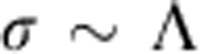
, the incoherent wave leads to the formation of several coherent DSWs, which develop within each individual fluctuation of 
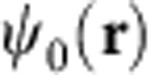
 as shown in Fig. 1c,d,f. Since the range of the nonlocal response is smaller than the coherence length 
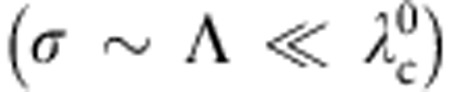
, every individual fluctuation evolves independently of each other. Hence, in this quasi-local (strong) turbulent regime, the incoherent wave develops singularities that are in essence coherent DSWs. These dispersive shocklets can be regarded as the conservative counterpart of viscous shocklets considered in high-speed turbulent flows^48^. Note that the development of a sea of DSWs manifests itself by a spectral filamentation process in frequency **k**-space (Fig. 1e). This is associated with the strong interaction among DSWs that emanate from different fluctuations and whose compression against each other leads to polygon-like patterns featured by effective one-dimensional (1D) DSW sides (Fig. 1d,f). Also note that although this shocklets regime occurs in the presence of wave dispersion (
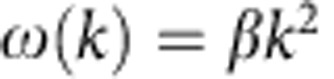
 from the linearized NLSE), it exhibits some interesting connections with a long-standing challenging issue of weakly dispersive acoustic-like wave turbulence^52^,^53^ (see Discussion section and Supplementary Note 4).

This physical picture changes in a dramatic way in the highly nonlocal regime, 
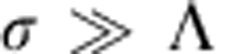
. This is illustrated in Fig. 1g–l, which shows the evolution of the field starting from the same initial condition 
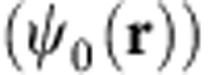
 as in the quasi-local regime. In the highly nonlocal regime, the fluctuations of the incoherent wave exhibit a global collective behaviour, which is responsible for the formation of a large-scale incoherent shock wave of a fundamental different nature. It is now the incoherent wave as a whole that develops a shock: the momentum of the speckled beam is radially outgoing (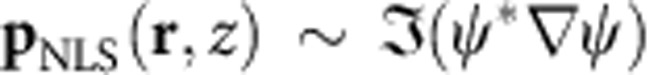 oriented along **r** as illustrated by the arrows in Fig. 1k) and exhibits a shock-like singularity, while the envelope of the intensity of the beam experiences an annular (ring-shaped) collapse-like behaviour (Fig. 1g–l). The fluctuations of the incoherent wave then result to be pushed towards the annular shock front, which leaves behind itself an internal region of the beam with a high degree of coherence. In other terms, the dynamics is featured by a dramatic degradation of the coherence properties on the annular boundary of the beam (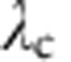 decreases with *z*), while its internal region exhibits a significant coherence enhancement (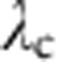 increases with *z*) (Fig. 1g–l).

We study this peculiar phenomenon through the analysis of the ‘local spectrum' (or spectrogram) of the random wave, that is, a spectrum that depends on the spatial position (**r**) because the random wave is characterized by fluctuations that are inhomogeneous in space. As illustrated in Fig. 1m–o, the evolution of the spectrogram exhibits a peculiar *Z*-shaped distortion, which contrasts with the regular deformation observed for 
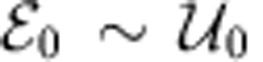
 (Supplementary Note 1 and Supplementary Fig. 2). In the strong nonlinear regime, 
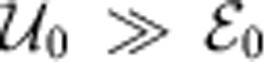
, the spectrogram exhibits a pronounced self-steepening that leads, nearby the overtaking point, to a dramatic spectral broadening on the annular boundary of the incoherent beam, where the coherence length decreases down to the healing length, 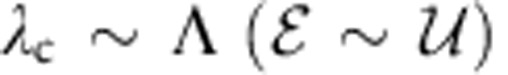. Here the increase in the linear energy 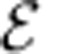 up to 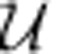 shown in Fig. 1p is due indeed to such peculiar process of coherence degradation. This is in marked contrast to coherent DSWs in Hamiltonian systems in which such a balance between linear and nonlinear energies stems from the well-known formation of regular DSW oscillations^24^,^33^,^34^,^35^,^36^,^38^,^39^,^40^,^41^,^42^,^43^,^44^,^45^.

### Singular solutions

We describe theoretically the phenomenon of large-scale incoherent shock within the general framework of the wave turbulence formalism. The wave turbulence theory has been shown to provide a natural asymptotic closure of the hierarchy of moment equations for a system of weakly nonlinear dispersive waves^3^,^4^,^5^. On the basis of a generalized wave turbulence approach of the problem, we show that the appropriate statistical description of the large-scale incoherent shocks is provided by a collisionless LRVE^14^,^55^. Note in this respect that resonant four-wave interactions described by a collision term are still present in the long-term evolution of the system, although in the long-range regime their influence on the dynamics results of higher order with respect to the LRVE dynamics, see Methods section.

**Figure 2:**
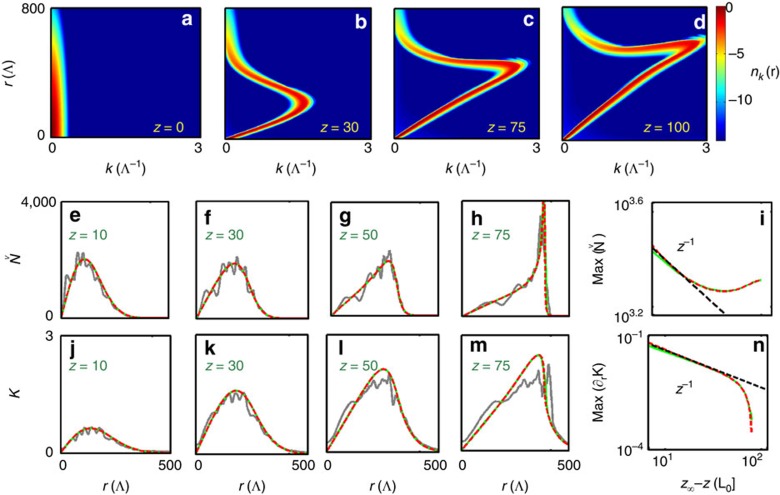
Incoherent shock and annular collapse singular behaviours. (**a–d**) Evolution during the propagation of the spectrogram 
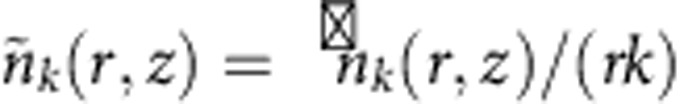
 obtained by solving the effective 1D LRVE (2). Evolutions of the intensity *Ň*(*r*, *z*) (**e**–**h**), and of the momentum *K*(*r*, *z*) (**j**–**m**), obtained by simulations of the NLSE (1) (grey), LRVE (2) (green), and ‘hydrodynamic' model (3–4) (dashed red). The annular collapse singularity, max_*r*_(*Ň*(*r*, *z*)) (**i**), and shock singularity, max_*r*_(*∂*_*r*_*K*(*r*, *z*)) (**n**), are in agreement with the power law predicted by the theory ∼(*z*_∞_−*z*)^−1^, where *z*_∞_ denotes the blow-up propagation length, see equation (5) (dark-dashed). The propagation length *z* is in units of *L*_0_. The NLSE simulation in **e**–**n** refers to the radial averaging of the results reported in Fig. 1j–l. The quantitative agreement between NLSE, LRVE and hydrodynamic models in **e**–**n** is obtained without using adjustable parameters (see Methods section).

The long-range Vlasov formalism discussed here differs from the traditional Vlasov equation describing random waves in hydrodynamics^8^,^15^,^16^, plasmas^27^ or optics, such as, for example, incoherent modulational instabilities^14^,^56^, or the collective clustering of incoherent solitons with inertial nonlinearities^57^. On the other hand, the structure of the LRVE is analogous to that describing systems of particles with long-range, for example, gravitational, interactions^50^,^51^. Contrary to conventional weak-turbulence approaches^1^,^2^,^6^,^7^,^58^, we show that the LRVE is valid beyond the weakly nonlinear regime of interaction: Simulations of incoherent shocks in the strong nonlinear regime 
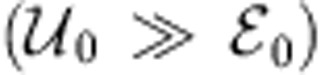
 reveal a quantitative agreement between LRVE and NLSE models without adjustable parameters (Fig. 2 and Supplementary Fig. 6).

We show in the Supplementary Note 2 that, if the initial condition is strongly nonlinear 
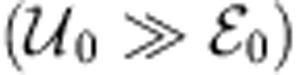
, the momentum of the incoherent wave results to be radially outgoing, so that the 2D LRVE remarkably reduces to an effective 1D equation:


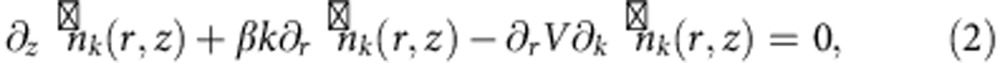


where *ň*_*k*_(*r*, *z*) is the local averaged spectrum of the field at radial position *r*=|**r**|, 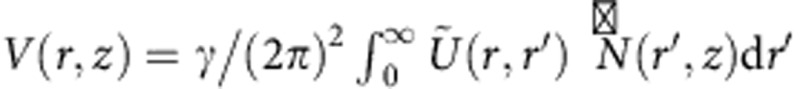, with 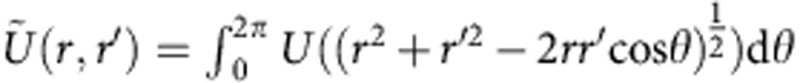 an effective radial response function and 
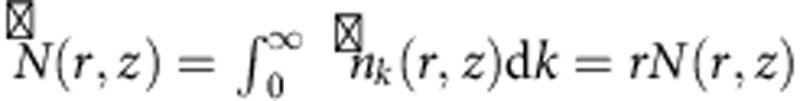
, see Methods section. A simulation of equation (2) is reported in Fig. 2a–d, which confirms the peculiar folding of the spectrogram discussed above through NLSE simulations in Fig. 1m–o. Physical insight into the strongly nonlinear regime can be obtained through solutions of equation (2) with extremely narrow spectral distribution, *ň*_*k*_(*r*,*z*)=*Ň*(*r*, *z*)*δ*(*k*–*K*(*r*, *z*)), where the ‘particle density' *Ň*(*r*, *z*) and ‘momentum' *K*(*r*, *z*) satisfy the following hydrodynamic-like model.


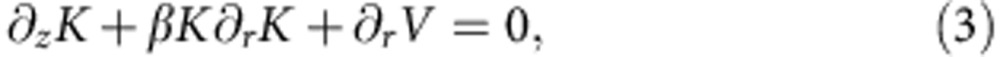



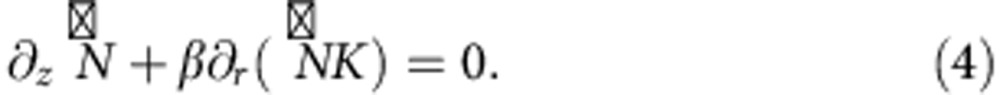


We remark that, from a broader perspective, the general class of singular solution considered here are sometimes called ‘mono-kinetic' (or single speed), in the sense that to each spatial position (*r*, *z*) corresponds a unique well-defined spectral (velocity) component (*k*, *z*) (ref. 51). This special form of singular solutions introduced in ref. 59 are important in that they make a direct correspondence with a generic hydrodynamic formalism, a property that found fundamental implications in a large variety of long-range interacting systems, for example, self-gravitating systems, 2D geophysical fluids, collisionless plasma or more generally wave–particle interactions^50^,^51^.

Numerical simulations of equations (3) and (4) are found in quantitative agreement with those of NLSE and LRVE equations, without using any adjustable parameter, as remarkably illustrated in Fig. 2e–n. Starting from *K*(*r*,*z*=0)=0, the ‘spectrogram' *K*(*r*, *z*) is first driven by the last nonlinear term in equation (3), while the Burgers-like (second) term of equation (3) subsequently leads to the gradient catastrophe of *K*(*r*, *z*). Quite remarkably, the finite ‘time' (distance, *z*) shock singularity of *K*(*r*, *z*) is responsible for an annular blow-up singularity of the intensity envelope *Ň*(*r*, *z*) on the circular boundary of the incoherent beam. These singular behaviours can be described theoretically by solving equations (3) and (4) by the method of the characteristics, which shows that the gradient of the spectrogram and the intensity envelope along a characteristic *R*(*z*) exhibit a blow-up singularity at the finite ‘time' *z*_∞_ (see Methods section):


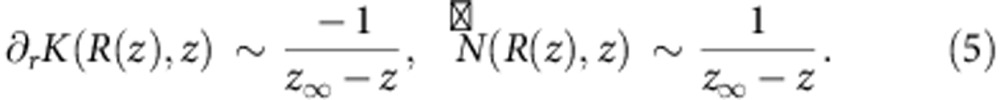


The tendency to diverge as ∼*z*^−1^ occurs regardless of the dimensionality of the problem that is, also in 1D (Supplementary Note 3 and Supplementary Figs 5 and 6), and has been confirmed by the simulations shown in Fig. 2i,n. Such dynamics becomes regularized by the NLSE and LRVE models, although an accurate description of the underlying regularization mechanism is hindered by a long-standing mathematical problem, namely, achieving a closure of the infinite hierarchy of *k*-moments equation for kinetic theories, see Methods section(Supplementary Note 3 and Supplementary Fig. 7).

We emphasize that the ‘hydrodynamic' model equations (3) and (4) recovers the 1D shallow-water equations under the substitution *V*(*r*, *z*)→*Ň*(*r*, *z*) (refs 33, 40). Note that, in the optical context, this limit is relevant to the description of incoherent wave propagation in inertial nonlinear media^14^,^56^,^57^. However, shallow-water equations are hyperbolic equations that are known to not exhibit collapse singularities: the annular collapse singularity of the field intensity in equations (3) and (4) originates into the nontrivial nonlocal coupling between the effective potential, 
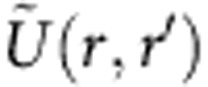
 and the ‘particle density', *Ň*(*r*, *z*) (see the expression of *V*(*r*, *z*) below equation (2)). Note that, in one spatial dimension, such a coupling reduces to a convolution, and the system still develops finite-time collapse and shock singularities (Supplementary Note 3 and Supplementary Figs 5 and 6).

### Experiments

**Figure 3:**
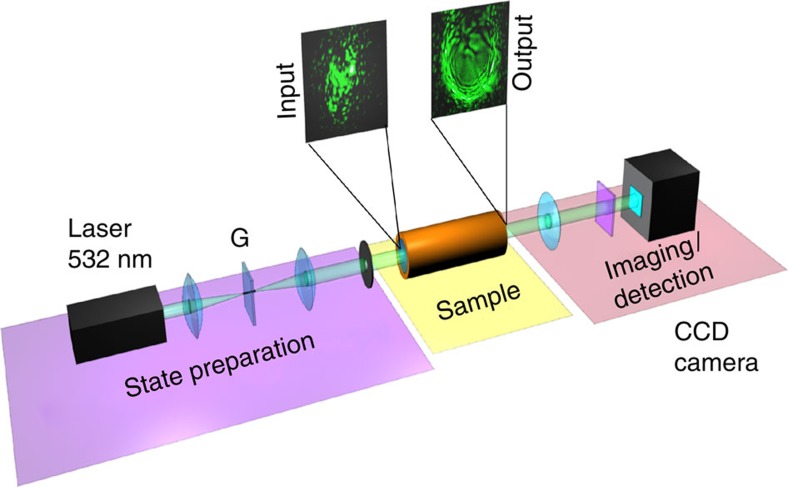
Experimental set-up. Experimental layout: the beam from a continuous wave (532 nm) laser is sent into the nonlinear material through a 4-f telescope. The sample consists of a cylindrical tube filled with a solution of methanol and graphene nanoscale flakes. A ground-glass plate (G) is placed in the focus of the first lens of the telescope to generate a speckle optical pattern with controlled correlation length, 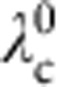 (see Methods section). After the sample, the beam is imaged onto a CCD camera.

We performed a series of experiments that provide evidence of both regimes of incoherent shocks and dispersive shocklets by varying the effective range of nonlocality in the nonlinear material. As illustrated in the experimental set-up in Fig. 3, the beam from a continuous wave laser (532 nm) is sent through a 4-f telescope. A ground-glass plate is placed in the focus of the first lens of the telescope, which creates a speckle pattern at the input of the sample. Notice that this initial speckle beam does not need to satisfy specific (for example, Gaussian) statistics because the incoherent shock phenomenon occurs irrespective of the wave statistics—a property justified by the fact that the LRVE formalism accurately describes the strongly nonlinear regime of interaction. The beam radius in the absence of the ground plate, and at the sample input window is 2.3 mm. The size of the speckles (that is, the coherence length of the random speckle pattern) at the sample input can be controlled by changing the beam size on the ground-glass plate, for example, by changing the beam size at the telescope input with an iris or, more simply, by shifting the position of the ground-glass plate along the beam propagation axis (Supplementary Note 1 and Supplementary Fig. 4). After the sample, the beam is imaged onto a CCD camera.

The liquid in the sample exhibits a thermal nonlocal nonlinearity that originates from a laser-induced heating of the liquid. The nonlinearity is intrinsically nonlocal due to heat diffusion and usually defocusing as a result of the negative thermo-optic coefficient (d*n*/d*T*<0). The nonlinear coefficient can be enhanced by including a highly absorbing dye (rhodamine^24^ or iodine). In our experiments, we chose a dilute solution of graphene nanoscale flakes that provides optimal conversion of absorbed laser energy into heat because of the absence of any fluoresence mechanisms. The medium exhibits very low absorption, which entails a highly nonlocal (long range) interaction, a property that has been exploited to study the incoherent shock phenomenon. The measured absorption coefficient is *α*=0.013 cm^−1^, so that the nonlocal length is in the range *σ*∼400 μm (ref. 24), an estimate that should be considered as lower bound, since in extended media, such as ours, the nonlocal length may be significantly larger, *σ*∼900 μm (Supplementary Note 1; ref. 22). Note that, despite the presence of absorption, the nonlinear system behaves essentially as a conservative (Hamiltonian) system, since the characteristic absorption length (≃76.9 cm) is much larger than the sample length (15 cm) and the nonlinear length (≃0.04 cm). The corresponding measured refractive index change is Δ*n*≃−2 × 10^−4^ at maximum input power (2.5 W) of the laser, which gives Λ∼5 μm. Considering typical values of the correlation length of the speckle beam, 
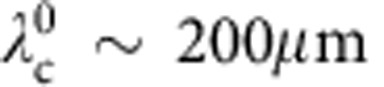
, we obtain the scale separation required for the observation of incoherent shocks, 
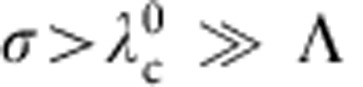
 (see Methods section and Supplementary Note 1 for more details).

**Figure 4:**
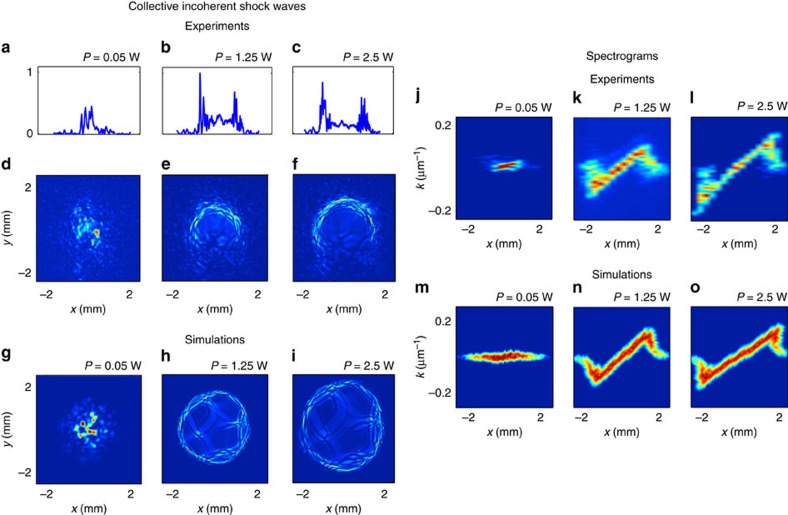
Experimental observation of incoherent shock waves (long-range regime). (**a**–**f**) Beam profiles of the output intensity taken at low power *P*=0.05 W (**d**), *P*=1.25 W (**e**) and *P*=2.5 W (**f**); corresponding intensity lineouts (at *y*=0) (**a**–**c**). Note that negligible linear diffraction occurs within the sample, so that the output profile in **d** in the linear regime (low power) also corresponds to the input profile at *z*=0. The asymmetry in the lower part of the beam is due to convection within the sample. (**g**–**i**) Numerical simulations of NLSE (equation (1)) performed with the experimental parameters (
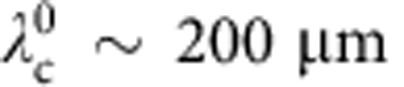
, Λ=4 μm, 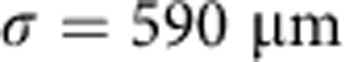). (**j**–**o**) Spectrogram signature of incoherent shocks: Experimental (**j**–**l**) and numerical (**m**–**o**), spectrograms retrieved from the lineouts *y*=0 (see Methods section). As the pump power increases, the spectrogram evidences the development of a shock singularity on the annular boundary of the beam, as predicted by the hydrodynamic model (3–4) (Fig. 2e–n).

Figure 4a–f reports typically measured output intensity patterns of the optical beam. As the input power is increased, we clearly see an annular reshaping of the speckled beam, with high-frequency components piling up on the boundaries, whereas low-frequency components dominate the internal region of the beam. This is confirmed by the corresponding *y*=0 lineouts in Fig. 4a–c, which evidence the formation of two lateral high-intensity peaks, as predicted by the collapse behaviour. The corresponding spectrogram measurements reported in Fig. 4j–l were obtained by measuring the far-field of the optical beam for several different *x*-positions. The singular distortion of the spectrograms provides a clear experimental signature of the phenomenon of incoherent shock. We also performed experiments in other liquids (for example, ethanol) and obtained similar results. Moreover, reducing the initial coherence length 
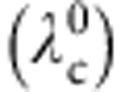
 leads to a substantial different dynamics with no evidence of incoherent shock formation (Supplementary Figs 1 and 2).

**Figure 5:**
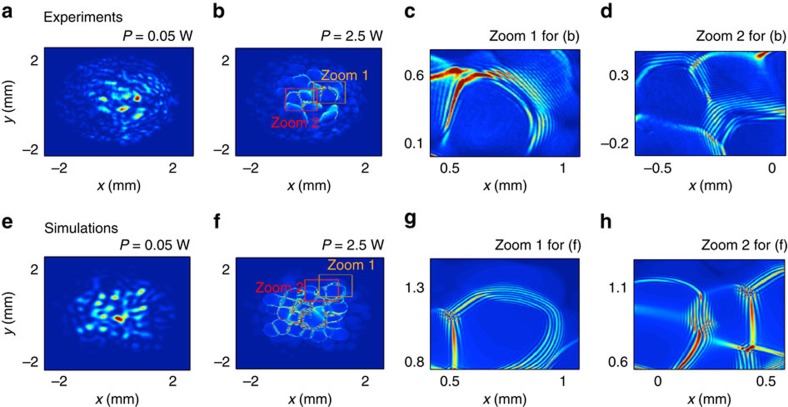
Observation of dispersive shocklets from a speckled beam (short-range regime). (**a**,**b**) Experimental beam profiles of the output intensity recorded at low power *P*=0.05 W (linear propagation) (**a**) and *P*=2.5 W (**b**). (**c**,**d**) Zooms on details of **b** that evidence the development of several DSW patterns. (**e**,**f**) Numerical simulations of NLSE equation (1) performed with the experimental parameters (
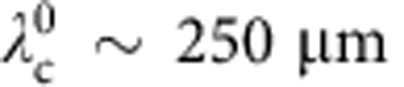
, Λ=3 μm, 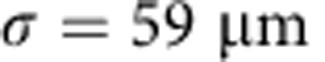), and corresponding zooms (**g**,**h**). As revealed by the zooms, a qualitative agreement is obtained between the experimental and numerical DSW periodic patterns. Note that simulations with 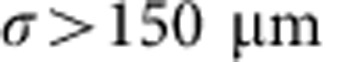 do not reveal the formation of DSWs, which confirms the effective short-range regime of interaction in the experiment (Supplementary Note 1).

The simulations of NLSE (1) have been performed by following the model developed in ref. 24, in which an effective 2D reduction of the three-dimensional (3D) heat equation is used to describe the thermal nonlinearity (Supplementary Note 1). Several works highlighted the role of remote boundary conditions of the thermal nonlinear sample in the dynamics of soliton propagation by considering the complete 3D heat equation^19^,^20^,^21^. In this respect, despite the fact that the nonlinear response function is in principle ‘infinite range', the analysis revealed that in actual experiments the response function can be accurately characterized by an effective well-defined finite range^22^. The 2D reduction of the heat equation considered here precisely accounts for such an effective finite nonlocal range, *σ* (refs 18, 24). In this way, we model the experiment by the universal form of the NLSE (1), in which the nonlocal response function does not depend on the propagation (time) variable, *z*, in the present case, *U*(*r*)*=K*_0_(*r*/*σ*)/(2*πσ*^2^), where *K*_0_(·) refers to the modified Bessel function of the second kind (Supplementary Note 1). It is interesting to note that, in spite of the simplicity of the 2D NLSE model (1) (for example, it does not account for heat-induced convection of the liquid, which is responsible for the up–down asymmetry in Figs 4 and 5), this model captures the essential phenomenological behaviour observed experimentally, as revealed by the qualitative agreement between the simulated intensity patterns (Fig. 4g–i and Fig. 5e–h) and spectrograms (Fig. 4m–o) with the experimental results.

In order to demonstrate that the emergence of such collective incoherent structure is ultimately linked to the long-range regime of interaction, we have made a second series of experiments to investigate the development of dispersive shocklets from a speckled beam. To this end, we significantly increased the concentration of graphene nano-flakes so as to increase the absorption (*α*≃1.7 cm^−1^), and thus reduce the nonlocal range of interaction by one order of magnitude, see Methods section. In order to inhibit long-range collective effects, the correlation length of the input speckle beam has to be chosen much larger than the nonlocal range, 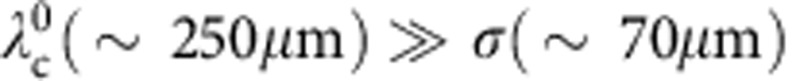. As illustrated in Fig. 5a–d, each individual speckle of the incoherent beam develops its own DSW, which leads to the formation of regular undular patterns, whose typical spatial period has been found in good agreement with the corresponding NLSE (1) simulations. As expected from the previous analysis, experiments realized with a smaller correlation length lead to an effective global behaviour characterized by a strong interaction among the different speckles, which strongly reduces the development of DSWs within each individual speckle of the input beam (Supplementary Fig. 3). For more details on the experimental results, in both short- and long-range regimes, and the corresponding NLSE simulations, see Supplementary Note 1.

## Discussion

We have reported a novel scenario of strongly nonlinear turbulent flows characterized by the emergence of a large-scale incoherent shock, which is inherently a collective phenomenon of the turbulent field as a whole. This strongly nonlinear turbulent regime is of a fundamental different nature than other forms of turbulence, which are dominated by nonequilibrium stationary cascades^1^,^2^,^6^,^7^,^8^,^12^, or deeply affected by localized nonlinear structures, such as solitonic turbulence^7^,^12^, or entangled vortices in superfluid turbulence^13^. By varying the range of the nonlocal thermal nonlinearity over one order of magnitude, a remarkable qualitative agreement has been obtained between the experimental results and the simulations of the NLSE model based on a 2D reduction of the heat equation. Despite such a qualitative agreement, it would be interesting to generalize the theory by considering the whole 3D Poisson equation describing heat diffusion also along the optical propagation axis^20^,^21^. This would lead to the unusual and interesting situation in which the long-range potential in the Vlasov formalism would depend itself on the ‘temporal' *z* variable, a property that would introduce intriguing unexplored collective behaviours of the turbulent flow.

We also briefly comment an interesting connection between the coherent shocklets regime reported here and a long-standing challenging issue inherent to weakly dispersive turbulent systems^52^,^53^. At variance with strongly dispersive wave systems that can be accurately described by the wave turbulence theory^1^,^2^,^3^,^4^,^5^,^6^,^7^, a proper kinetic formulation of random nonlinear waves that exhibit weak acoustic-like dispersion constitutes a difficult problem that has been the subject of important developments in the past^1^,^2^,^52^,^53^. The first serious attempt of an analytical description of a ‘semi-dispersive' wave turbulence was reported in ref. 52. Basically, the theory accurately describes how energy is shared along ‘rays' in the frequency domain, that is, between wave vectors with the same orientation in **k**-space; however, it does not describe how energy is redistributed between distinct neighbouring rays. The foundation for an evaluation of the contribution to energy exchange that occurs at higher orders was formulated theoretically in ref. 53. This leaves unanswered an open challenging issue^2^,^53^: Given an initial anisotropic spectral distribution of the weakly dispersive random wave, one may wonder whether the nonlinear interactions of the next order lead to a spectral isotropic redistribution or to condensation along specific rays in **k**-space. In the light of this problem, we have performed NLSE simulations in the presence of weak dispersion (Bogoliubov regime), which reveal that, locally, the spectrum can exhibit some remarkable effects of spectral star formation and ray condensation in **k**-space, although their properties and underlying mechanisms depend on the nature of the random fluctuations as well as the dispersive properties of the waves (Supplementary Note 4 and Supplementary Figs 8–13). These preliminary simulations open an array of interesting questions, which will be considered in future works specifically devoted to this important issue of acoustic turbulence.

From a broader perspective, our photon fluid system can be viewed as a general platform for the experimental study of this acoustic turbulent regime. Furthermore, thanks to its widely tunable degree of nonlocality, it also opens the door to the experimental study of a variety of collective incoherent wave phenomena. We briefly comment here the applicability to extreme wave events, also called rogue, killer or freak waves^15^,^46^,^60^. Although these phenomena have been shown to emerge from a turbulent state, so far, the rogue wave itself has been always considered as being inherently a coherent localized entity. Here, by considering the shock and collapse singularities in the focusing regime 
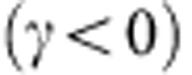
, we may expect that the incoherent wave as a whole would lead to the formation of an extreme high-amplitude event, a feature that would be interpreted in analogy with an incoherent rogue wave phenomenon. Works are in progress in order to study the existence and the spontaneous emergence of this novel form of extreme events from a turbulent state of the field. The experimental study requires a highly nonlocal focusing nonlinearity, while usual pure liquids exhibit a defocusing thermal nonlinearity. Interestingly, however, a focusing thermal nonlinearity can be achieved by exploiting the Sôret effect in nanoparticle suspensions, which would thus couple the nonequilibrium behaviour of the turbulent field with the thermophoretic motion of the particles in the liquid^30^,^31^. Along these general lines, it would also be interesting to consider the relevance of the global collective phenomenological behaviours reported here within the general framework of opto-fluidics^61^. In this respect, while optical manipulation in strongly scattering densely packed (for example, biological) suspensions is usually considered impossible, a recent work demonstrated that some controlled manipulation of opaque suspensions can be achieved through the formation of a large-scale shock of nanoparticle concentration^62^. The long-range kinetic formalism developed here could be relevant to shed new light on the complex nonlinear mechanisms, which underlie this interesting example of highly nonlocal and diffusive opto-fluidic system.

Furthermore, considering the universality of the NLSE, these collective nonequilibrium behaviours find applications in a multitude of disciplines in nonlinear science. Moreover, such large-scale incoherent singularities can also develop in the temporal domain, thanks to a noninstantaneous (instead of nonlocal) response function. At variance with spatial nonlocality, temporal nonlocality is characterized by a response function that is constrained by the causality condition^14^,^63^, which breaks the Hamiltonian structure, and thus introduces collective incoherent shocks and collapses phenomena of a different nature than those reported here. Then in addition to nonlocal wave systems discussed above through (refs 17, 18, 19, 20, 23, 24, 25, 26, 27), our work is also relevant to many physical systems involving a radiation–matter interaction featured by a finite-time nonlinear response.

## Methods

### Formalisms

The NLSE (1) conserves the power 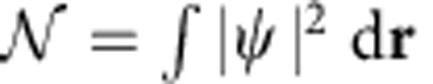, and the Hamiltonian, 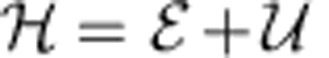, which has a linear contribution 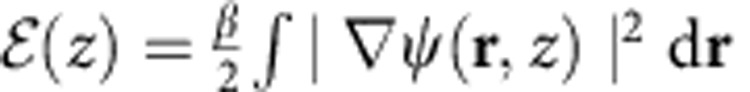, and a nonlinear contribution, 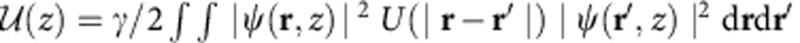. The LRVE describes the evolution of the averaged local spectrum of the wave: In two dimensions (*D*=2) it reads, 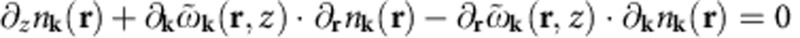, where the local spectrum, *n*_**k**_(**r**, *z*), is the Wigner-like transform of the correlation function, 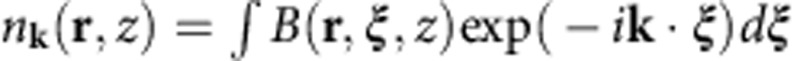 with 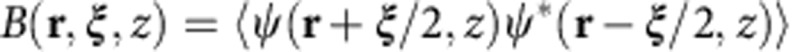 and 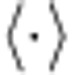 denotes an average over the realizations^14^. The generalized dispersion relation is 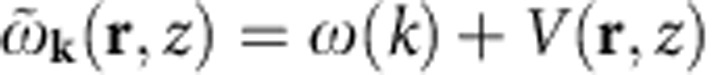, where 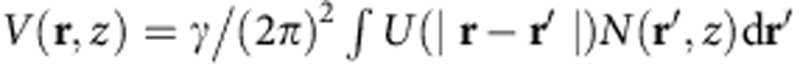 is the effective potential, 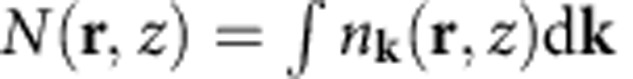 is the envelope intensity, and 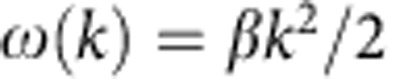.

Assuming initial radial symmetry, the general solution of the LRVE depends on *r*=|**r**|, *k*=|**k**| and the angle cos*θ*=**k**·**r**/(*kr*), namely, *n*_**k**_(**r**, *z*)=*n*_*k*_(*r*, *θ*, *z*). However, we show in Supplementary Note 2 that, if the initial condition is strongly nonlinear 
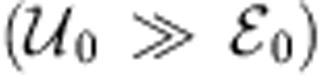
, the solution of the 2D LRVE takes the form 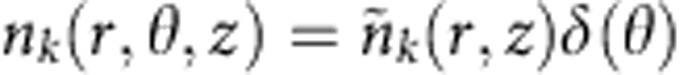 for *z*>0, that is, the local momentum is radially outgoing, 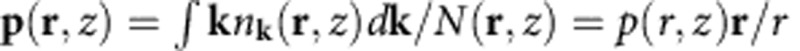. It turns out that the dynamics is described by the effective 1D LRVE (2) for the local spectrum 
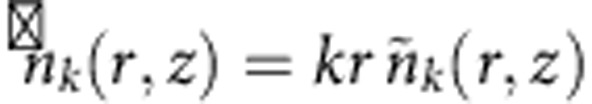
, where 
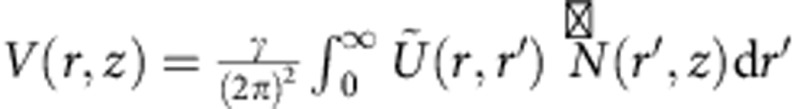
, with 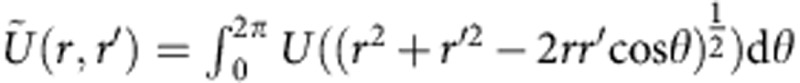, 
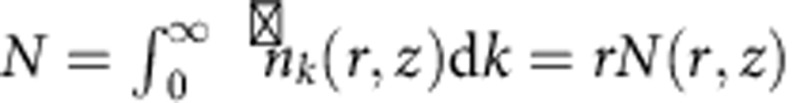
, and 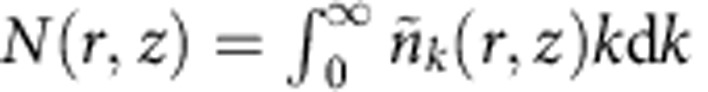 (Supplementary Note 2).

The momentum, *K*(*r*, *z*), in Fig. 2j–n has been calculated as follows: for the NLSE, *K*_NLS_(*r*, *z*)=**p**_NLS_(**r**, *z*)·**r**/*r*, where 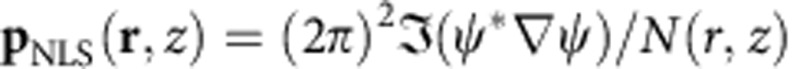 refers to the local momentum of the stochastic field 
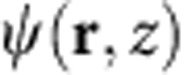
; for the LRVE, 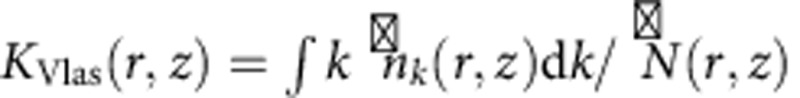. The simulations in Figs 1 and 2 have been performed with a Gaussian-shaped response function, 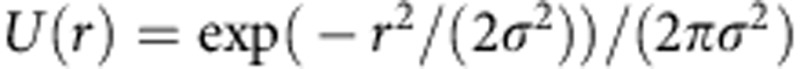.

In order to compare the simulations of the NLSE and LRVE models, we normalized the fields with respect to the size of the numerical window (*L*=1,200Λ in Figs 1 and 2). As discussed in the main text, a quantitative agreement has been obtained between NLSE and Vlasov simulations even in the strongly nonlinear regime. As far as we know, there is no rigorous proof of the validity of the LRVE in the strongly nonlinear regime. This fact may be qualitatively interpreted by using arguments similar to those of refs 55, 64, in which it was shown that Gaussian statistics is preserved during the nonlinear evolution of the system.

### Influence of the collision term on LRVE

The simulations reveal that in the highly nonlocal (that is, long range) regime of interaction, 
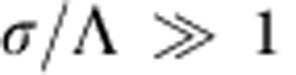
, the dynamics of a random wave characterized by a non-homogeneous statistics is dominated by the LRVE, which indicates that the wave turbulence collision term is of higher order. This can be interpreted by considering the following collision term in the rhs of the LRVE, 
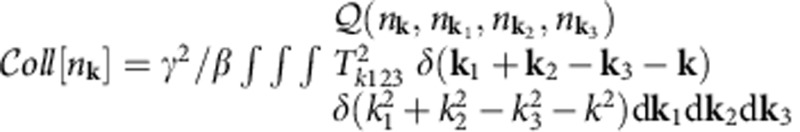
 where 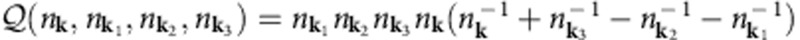 while the tensor *T*_*k*123_ accounts for the nonlocal interaction^1^,^6^,^14^, 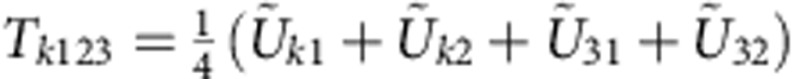, with 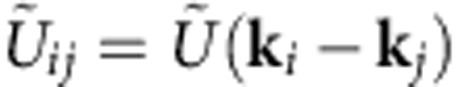, and 
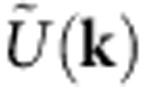
 the Fourier transform of the response function *U*(**r**). A qualitative analysis of the LRVE reveals that the typical nonlinear propagation length scale is of the order 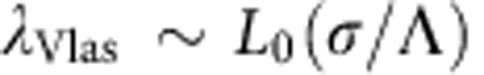 (ref. 65). On the other hand, the collision term slows down the dynamics by a factor of order equal to or larger than 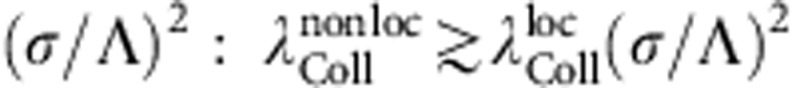 (ref. 14), where 
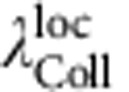
 is the nonlinear propagation length scale of the local collision term (*U*(**x**)→*δ*(**x**), and thus *T*_*k*123_=1). Considering a mode of the order *k*∼1/Λ, we have typically 
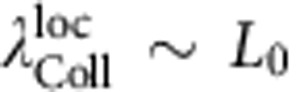
, so that 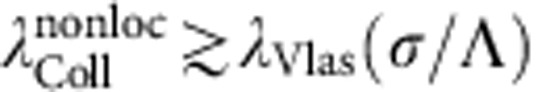. Then in the long-range regime, 
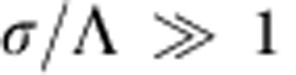
, the collision term is of higher order with respect to LRVE (note that 
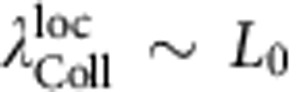
 should be considered as a lower bound, since 
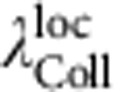
 increases for higher modes *k*>>1/Λ required to verify the weakly nonlinear regime of interaction, 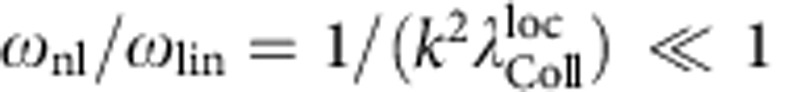 (ref. 3)). In the highly nonlocal configuration of the experiment, we have 
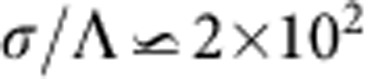
 (Fig. 4), which indicates the irrelevance of the collision term to describe the experimental results in the long-range regime. Note that in the limit of a local interaction, the generalized collisional Vlasov equation recovers the form of the kinetic equation recently considered in ref. 8 to study the stability of Kolmogorov–Zakharov spectra of turbulence.

### Finite ‘time' singularities

We made use of the method of the characteristics to solve analytically the ‘hydrodynamic' model (3–4). The quantities *w*(*z*)=*K*(*R*(*z*), *z*), 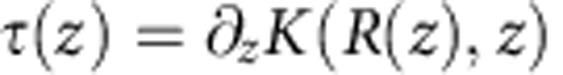, 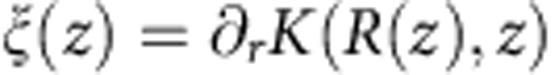, and 
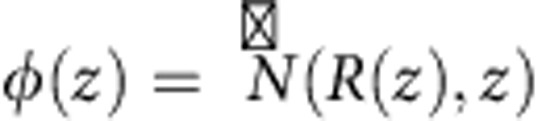
 can be shown to satisfy: 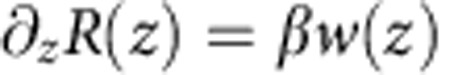 with *R*(0)=*r*_0_; 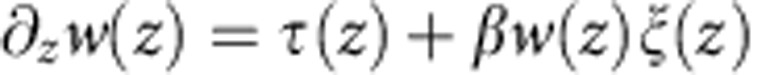 with *w*(0)=0, while


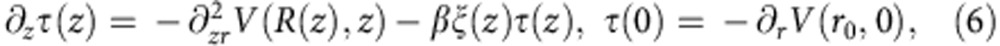



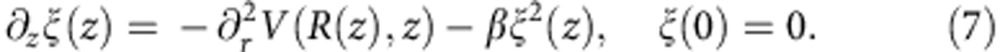



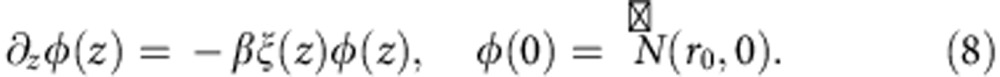


We note that *V*(*r*, *z*) and its *r*-derivatives are uniformly bounded: 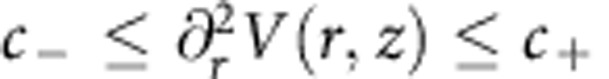, where 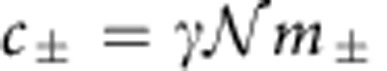
with 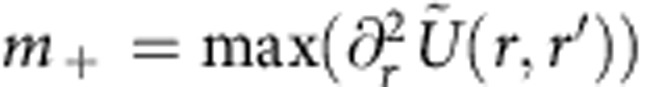 and 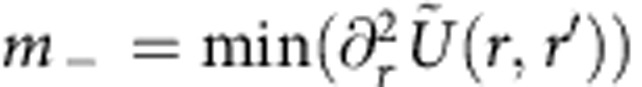. If *c*_≥0, then 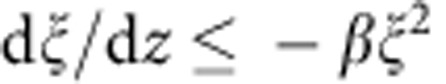 and 
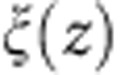
 blows up in finite ‘time'. If *c*_<0, then 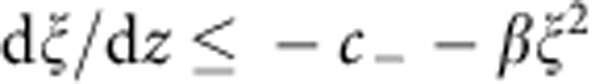, so that whenever 
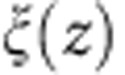
 reaches 
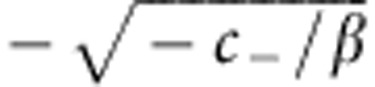
, then 
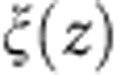
 also blows up in finite ‘time'. Remarking furthermore that 
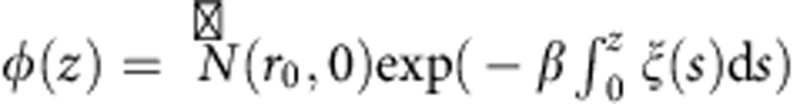
 from equation (8), we obtain the singular behaviours of 
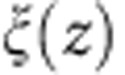
 and 
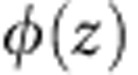
 given in equation (5) just before *z*=*z*_∞_: 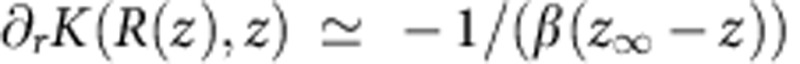, *N*(*R*(*z*), *z*)≃*r*_0_*N*(*r*_0_, 0)/(*R*_∞_(*z*_∞_–*z*)), while *w*(*z*) and *R*(*z*) converge to some finite limits, *w*(*z*)→*w*_∞_, *R*(*z*)→*R*_∞_. Note that the analysis can be extended to the focusing regime 
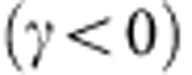
, where the singularity occurs at the centre of the beam (*r*=0).

### Regularization and closure

The finite ‘time' singularities (5) described by the hydrodynamic model (3–4) are regularized by the NLSE and LRVE equations. The spectrogram 
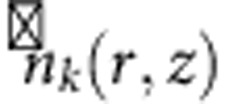
 evolves in the 2D phase-space (*r*, *k*) and can thus become ‘multi-valued' (Fig. 2 and Supplementary Fig. 7). However, the derivation of reduced equations describing the regularization of the singularities (5) is a difficult task fundamentally related to a long-standing mathematical problem, namely achieving a closure of the infinite hierarchy of equations that govern the evolutions of *k*-moments in transport kinetic equations. Note that the wave turbulence closure is justified in the weakly nonlinear regime^1^,^2^,^6^,^7^, while the closure considered here concerns the opposite strongly nonlinear regime. We address this problem of regularization in the Supplementary Note 3 through the analysis of higher-order truncations of the hierarchy (Supplementary Fig. 7). Our study reveals that all higher-order *k*-moments suddenly become of the same order of magnitude nearby the singularities (5), which prevents a closure of the infinite hierarchy and thus a reduced description of the dynamics beyond the incoherent shock point. Also note that in the context of self-gravitating systems, a weak diffusive effect has been introduced in a pure phenomenological way in order to regularize the wave breaking shock singularity described by the inviscid Burgers equation, thus leading to the so-called ‘adhesion model'^66^. To our knowledge, no rigorous theory has been developed to justify such an heuristic approach^51^.

### Experiments

For the incoherent shock experiment, Fig. 4, the sample is a 15-cm-long, 2-cm-diameter cylindrical tube, with anti-reflection (at 532 nm)-coated windows, and is filled with a solution of methanol and graphene nano-flakes. The graphene flakes have strongly subwavelength dimension (an average thickness of 7 nm, Graphene Supermarket) and thus do not induce significant scattering of light. They do, however, induce additional absorption and then release the absorbed energy to the surrounding liquid in the form of heat—less than 20% measured absorption over the whole sample length has been measured 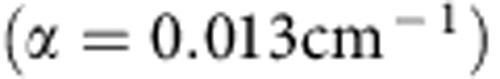. For the dispersive shocklets experiment (Fig. 5), the concentration of graphene flakes has been increased (Δ*n*≃−4 × 10^−4^) so as to increase absorption 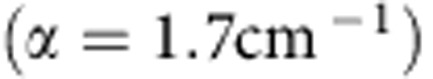, and thus reduce the nonlocal range. Note that, due to such a strong absorption, the optical power transmitted through the sample is significantly reduced in the dispersive shocklets experiment, so that the sample length has been reduced to 1 cm. Although light absorption during propagation is no longer negligible, its impact on the dynamics is still perturbative with respect to nonlinear effects, 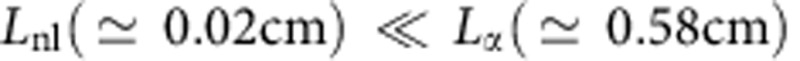. According to the scaling 
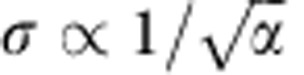
 (ref. 24), the nonlocal range has been reduced by one order of magnitude in the coherent shocklets experiment as compared with the incoherent shock experiment. This important reduction of the nonlocal range has been confirmed by a direct comparison between the experiments and the NLSE simulations, in which the precise value of 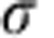 has been adjusted to reproduce the experimental results: A minimum value of 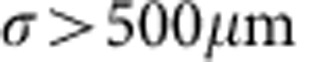 is required in order to simulate the collective behaviour of the incoherent shock experiments, while accurate simulations of the dispersive shocklets experiments require a significant reduction of the nonlocal length, typically 
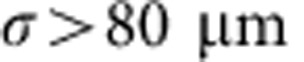
 (Supplementary Note 1 and Supplementary Figs 1–3). The measurement of the experimental spectrogram (*x*–*k*_*x*_) in Fig. 4j–l has been performed by recording the far-field spectrum of the optical beam for several different *x*-positions and by keeping fixed the *y*-position and the pump power. For more details on the experimental results and corresponding numerical NLSE simulations, see Supplementary Note 1.

### Data availability

All relevant experimental data present in this publication can be accessed at doi: 10.17861/fd97bbaa-af4f-4ce2–818c-dfb032b5229c.

## Additional information

**How to cite this article:** Xu, G. *et al*. From coherent shocklets to giant collective incoherent shock waves in nonlocal turbulent flows. *Nat. Commun.* 6:8131 doi: 10.1038/ncomms9131 (2015).

## Supplementary Material

Supplementary Information Supplementary Figures 1-13, Supplementary Notes 1-4 and Supplementary References
