# Supplementary material for: From coherent shocklets to giant collective incoherent shock waves in nonlocal turbulent flows
Source: Nat Commun. 2015 Sep 8;6:8131. doi: 10.1038/ncomms9131 (PMC4569716; doi:10.1038/ncomms9131)
Supplement: Supplementary Information — Supplementary Figures 1-13, Supplementary Notes 1-4 and Supplementary References [file ncomms9131-s1.pdf]

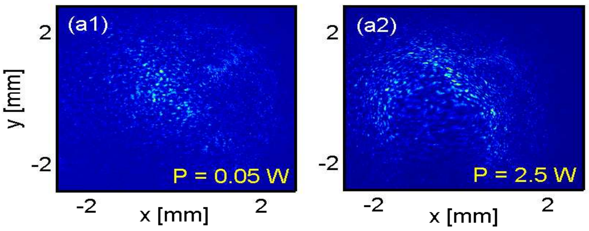

Supplementary Figure 1: **Additional experimental results in the incoherent shock regime.** The experimental configuration and corresponding parameters are the same as in Fig. 4 in the main paper, except that the correlation length of the input speckle beam has been decreased to $\lambda_c^0 \sim 70\mu$m. Intensity patterns recorded at the output of the nonlinear sample (15cm long) with a power P=0.05W (linear regime) (a), and P=2.5W (b). By reducing the input correlation length, λ_c^0, the dynamics slows down significantly and the spectrogram exhibits a regular deformation (see 2), which prevents the observation of the incoherent shock phenomenon.

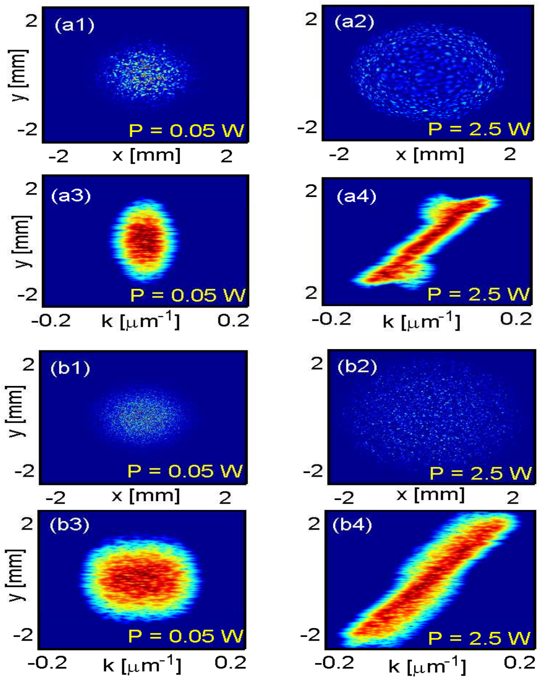

Supplementary Figure 2: **Simulations in the incoherent shock regime.** The NLSE simulations have been performed with the experimental parameters given in the text [after Eq.(1-2)], in particular $\alpha = 0.013\mathrm{cm}^{-1}$, $\sigma = 590\mu$m, $\Lambda = 4\mu$m and 15cm of propagation. In panels (a), the initial correlation length is of the same order as in the experimental result reported in Fig. 1 ($\lambda_c^0 \sim 70\mu$m), while in panels (b) the correlation length is reduced to $\lambda_c^0 \sim 20\mu$m. (a1) Intensity pattern at low power P=0.05W (linear regime) and $\lambda_c^0 \sim 70\mu$m, (a2) high power P=2.5W, (a3-a4) corresponding spectrograms. (b1) Intensity pattern at low power P=0.05W (linear regime) and $\lambda_c^0 \sim 20\mu$m, (b2) high power P=2.5W, (b3-b4) corresponding spectrograms. As the correlation length is reduced, the dynamics slows down and prevents the observation of the incoherent shock processes, as revealed by the regular deformation of the spectrogram evolution (b4), which contrasts with the peculiar $Z-$shaped distortion discussed in the main paper (Fig. 4).

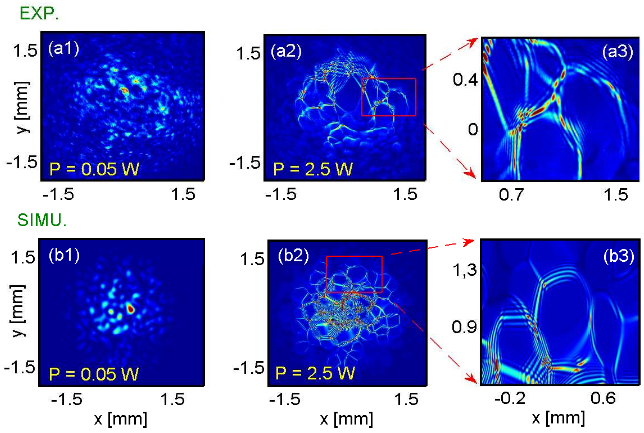

Supplementary Figure 3: **Experimental and numerical results in the dispersive shocklets regime.** The experimental configuration and corresponding parameters are the same as in Fig. 5 in the main paper, except that the correlation length of the input speckle has been decreased to $\lambda_c^0 \sim 110\mu$m. (a) Experimental results: intensity pattern recorded at the output of the sample at low power P=0.05W (linear regime) (a1), at high power P=2.5W (a2), zoom on a detail (a3). (b) Corresponding NLSE simulations: intensity pattern at low power P=0.05W (linear regime) (b1), and at high power P=2.5W (b2), zoom on a detail (b3). The simulations have been performed with the experimental parameters given in the text [after Eq.(1-2)], in particular $\alpha = 1.7$cm^{-1}, $\sigma = 59\mu$m, $\Lambda = 3\mu$m (1cm of propagation). These results can be compared to Fig. 5 in the main paper where $\lambda_c^0 \sim 250\mu$m: As the input correlation length decreases, the system exhibits an effective global behavior characterized by a strong interaction among the different speckles (see the big holes of low-intensity regions in (a2) and (b2)), which strongly reduces the development of dispersive shock waves within each individual speckle of the input beam. Note that the up-down asymmetry in the experimental DSW patterns (a2) can be ascribed to the presence of convection in the sample.

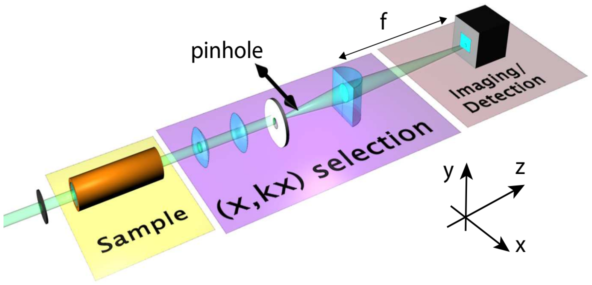

Supplementary Figure 4: **Set-up for spectrogram measurements.** Detail of experimental layout used to measure the x, k_x spectra in the main paper. We image the sample output with a 4-f telescope. In the imaging plane, we select an (x, y) circular section of the beam with a 0.5 mm diameter pinhole. This pinhole is translated laterally along the x-direction (with fixed y-direction). For each position of the pinhole the k_x spectrum is measured with a CCD camera placed at distance f=25 cm (focal distance) from a cylindrical lens.

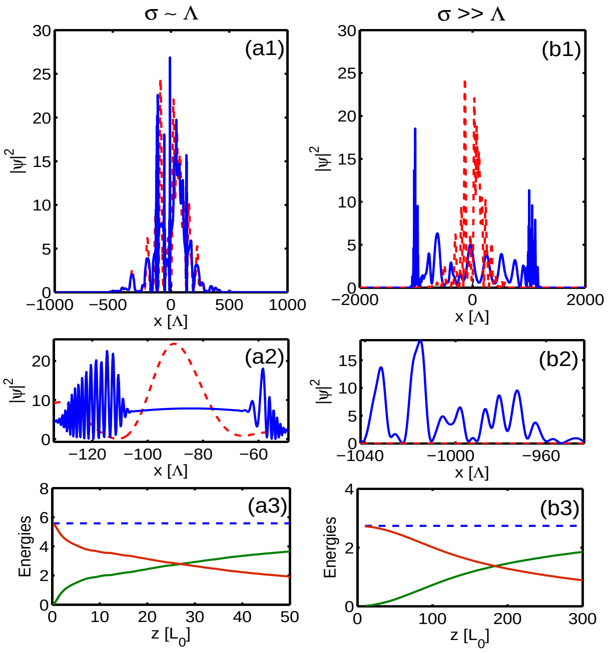

Supplementary Figure 5: **Incoherent shock waves in the defocusing one-dimensional case.** Numerical simulations of the NLSE (18) starting from the same initial condition, $\psi_0(x)$: (a1) In the quasi-local regime $\sigma = 2\Lambda$, each individual fluctuation of the incoherent wave develops a coherent DSW, as illustrated in the zoom (a2). (b2) In the highly nonlocal regime $\sigma \gg \Lambda$, the random wave as a whole develops an incoherent shock, which is characterized by a dramatic coherence degradation at boundaries of the incoherent structure (see (b2) for a zoom), while a significant coherence enhancement occurs within the structure. Panels (a3) and (b3) show the corresponding evolutions of $\mathcal{E}(z)$ (green), $\mathcal{U}(z)$ (red), while the total Hamiltonian $\mathcal{H} = \mathcal{E} + \mathcal{U}$ =const (dashed-blue): The regularizations of the ensemble of DSWs (left column), as well as the incoherent shock (right column), are characterized by an increase of the kinetic energy $\mathcal{E}(z)$ up to the nonlinear energy $\mathcal{U}(z)$.

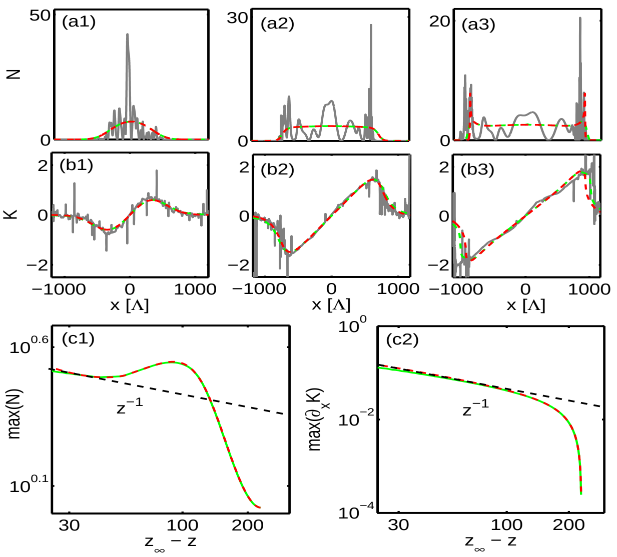

Supplementary Figure 6: **Agreement between NLSE, LRVE and 'hydrodynamic' models in the defocusing $1D$ case.** Evolutions of $N(x,z)$ (a) and $K(x,z)$ (b) obtained by simulations of NLSE (18) (gray), LRVE (19) (green), 'hydrodynamic' model (26-27) (dashed red), $z = 50, 160, 230L_0$ from left to right. The particle fluxes are evaluated as follows: for NLSE, $K_{\mathrm{NLS}}(x,z) = \frac{2\pi}{N}\Im(\psi^*\partial_x\psi)$, for the LRVE, $K_{\mathrm{Vlas}}(x,z) = \frac{1}{N}\int kn_k dk$. Corresponding evolutions of $\max_x\big(N(x,z)\big)$ (c1), and $\max_x\big(\partial_x K(x,z)\big)$ (c2), the dark-dashed line is the predicted power law z^{-1} from Eq.(28-29). The quantitative agreement is obtained without using any adjustable parameter.

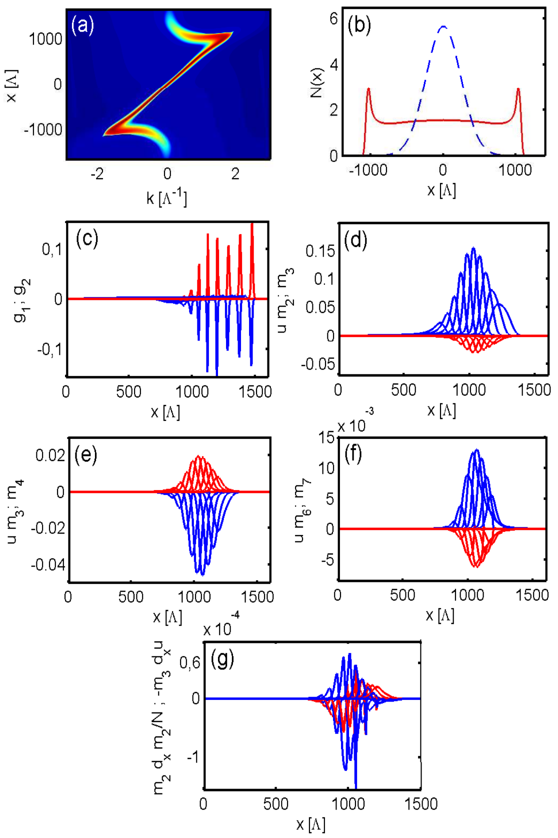

Supplementary Figure 7: **Impossibility of achieving a closure of the hierarchy of moments equations.** Comparison of different contributions of the moments from Eqs.(22-24). Numerical simulation of the LRVE (19) (in the main paper): Spectrogram $n_k(x, z_0)$ for $z_0 = 320L_0$ (a), and corresponding intensity distribution $N(x, z_0)$ in red (the dashed blue line shows the initial condition) (b). The solution of the LRVE, $n_k(x, z)$, has been used to calculated the following quantities: (c) Evolutions of $g_1(x, z) = u\partial_x p$ (green), $g_2(x, z) = -\partial_x m_2/N$ (blue). (d) Evolutions of $p(x, z)\, m_2(x, z)$ (blue), $m_3(x, z)$ (red); (e) Evolutions of $p(x, z)\, m_3(x, z)$ (blue), $m_4(x, z)$ (red); (f) Evolutions of $p(x, z)\, m_6(x, z)$ (blue), $m_7(x, z)$ (red); (g) Evolutions of $m_2(x, z)\partial_x m_2(x, z)/N(x, z)$ (blue), $-m_3(x, z)\partial_x p(x, z)$ (red). (c-g): from left to right: $z = 220L_0$ to $z = 400L_0$, with $\Delta z = 20L_0$, the incoherent shock occurs at $z_s \simeq z_0 = 320L_0$. The higher-order moment $m_{q+1}(x, z)$ becomes of the same order as $p(x, z)\, m_q(x, z)$ in the lhs of Eq.(24) [in the same way, $m_{q-1}(x, z)\partial_x m_2(x, z)/N(x, z)$ becomes of the same order as $m_q(x, z)\partial_x p(x, z)$], which prevents a closure of the infinite hierarchy of moment equations.

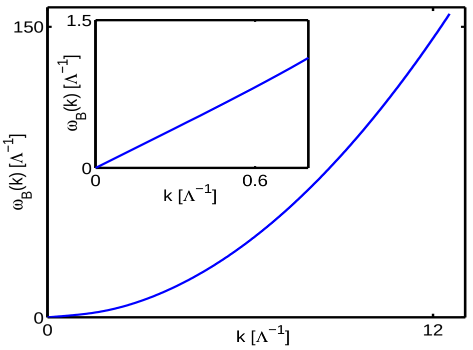

Supplementary Figure 8: **Acoustic-like dispersion relation.** Bogoliubov dispersion relation, $\omega_B(k)$, for a homogeneous wave amplitude $n_0 = 0.9$, a Gaussian nonlocal response function, $U(r)$, and a nonlocal range $\sigma = 2$ (in units of the healing length, Λ). As illustrated in the inset, typically for $|k| < 0.8$, the dispersion relation is acoustic-like, $\omega_B(k) \propto k$.

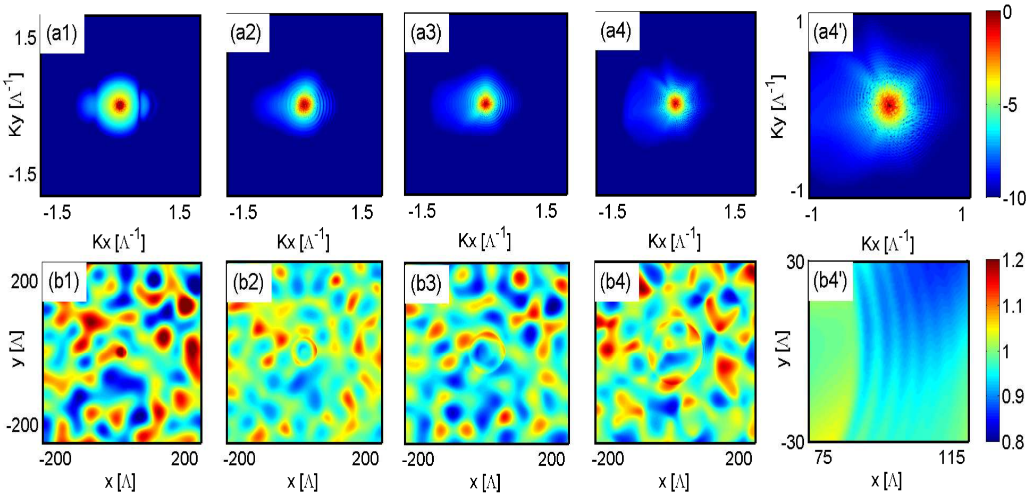

Supplementary Figure 9: **Asymmetric strong spatial fluctuation.** Evolution of the spectrum (a), and intensity (b), of a random wave characterized by the presence of strong asymmetric spatial fluctuation at $\mathbf{r} \simeq 0$, for $z = 0$, $z = 15$, $z = 25$, $z = 45$ from left to right – the last column (a4',b4') reports zooms corresponding to $z = 45$, note in particular the DSW in (b4'). The inhomogeneous spectral broadening ($z = 15$) exhibits an isotropic spectral homogenization ($z = 25$), which is then followed by a process analogous to 'RC' along specific rays in $\mathbf{k}-$space ($z = 45$). Note that the random wave evolves in the presence of a strong homogeneous (plane-wave) condensate, see the color-bar in (b). The spectra have been plotted in $\log_{10}$-scale by removing the plane-wave condensate spectral peak at $\mathbf{k} = 0$.

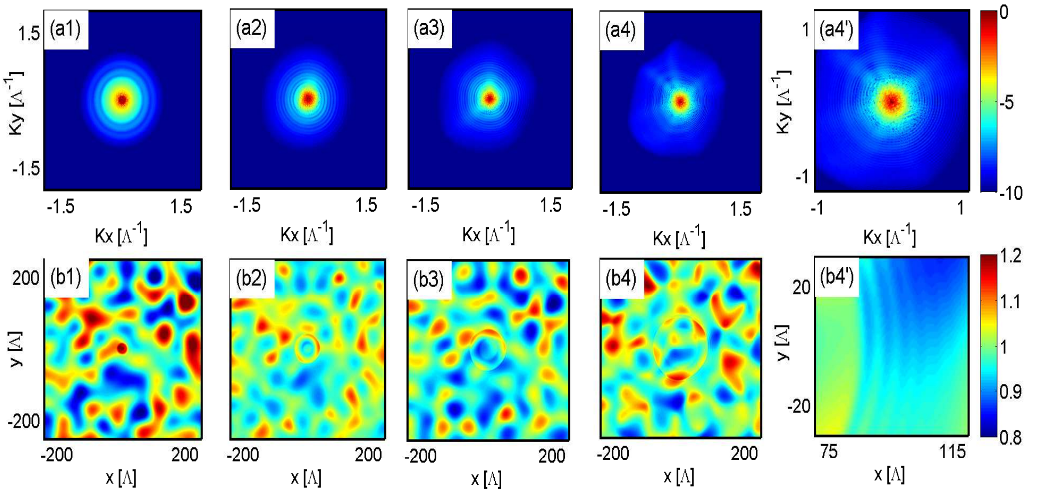

Supplementary Figure 10: **Symmetric strong spatial fluctuation.** Same initial condition as in Fig. 9, except that the asymmetric strong fluctuation at $\mathbf{r} \simeq 0$ has been replaced by a fully symmetric fluctuation of the same typical amplitude. From left to right: $z = 0$, $z = 15$, $z = 25$, $z = 45$ – the last column (a4',b4') reports zooms corresponding to $z = 45$, note in particular the DSW in (b4'). At variance with Fig. 9, the shock-induced spectral broadening of the strong fluctuation now occurs homogeneously, in both $\mathbf{r}-$space and $\mathbf{k}-$space, see (a2) and (b2). As a result, the 'RC' process leads to the formation of a 'star' in $\mathbf{k}-$space. Note that, as in Fig. 9, the random wave evolves in the presence of a strong homogeneous (plane-wave) condensate, see the color-bar. The spectra have been plotted in $\log_{10}$-scale by removing the plane-wave condensate spectral peak at $\mathbf{k} = 0$.

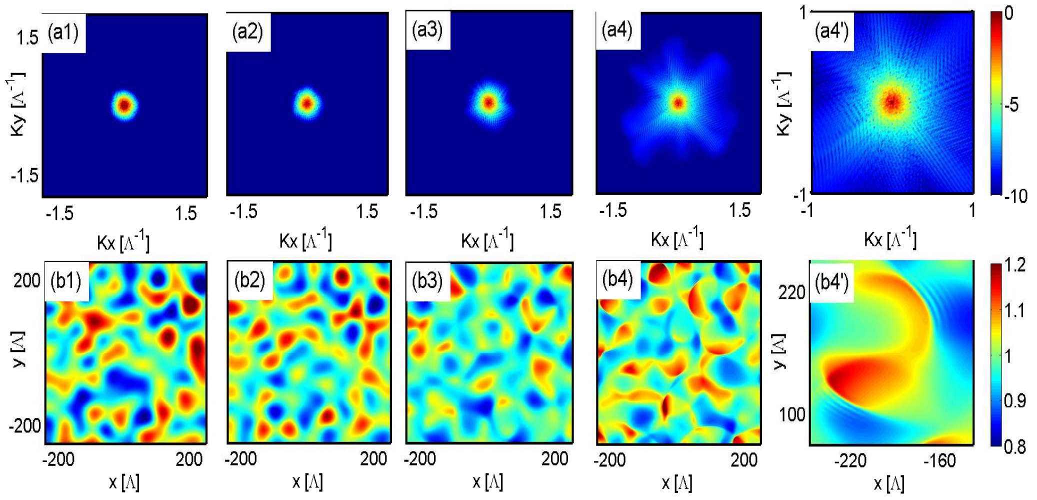

Supplementary Figure 11: **Without strong fluctuation.** Same initial condition as in Fig. 9 (or Fig. 10), except that the strong fluctuation at $\mathbf{r} \simeq 0$ has been removed. From left to right: $z = 0$, $z = 25$, $z = 45$, $z = 85$ – the last column (a4',b4') reports zooms corresponding to $z = 85$, note in particular the DSW in (b4'). In contrast to Figs. 9-10, the spectrum does not exhibit 'RC' at $z = 45$, while a 'star' in $\mathbf{k}-$space occurs at a later propagation length, $z = 85$. The random wave evolves in the presence of a strong homogeneous (plane-wave) condensate, see the color-bar. The spectra have been plotted in $\log_{10}$-scale by removing the plane-wave condensate spectral peak at $\mathbf{k} = 0$.

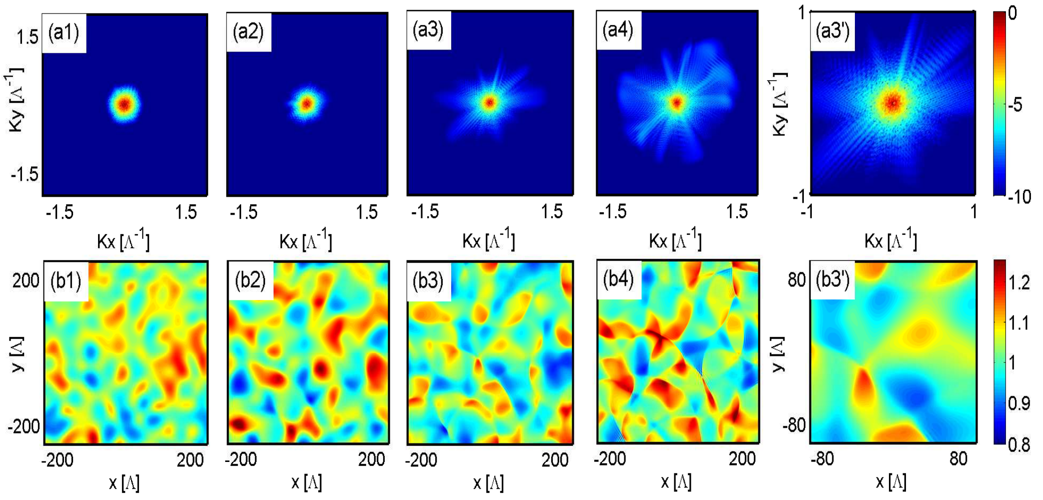

Supplementary Figure 12: **Without strong fluctuation (closely spaced speckles).** Initial condition similar to that of Fig. 11, except that the speckles fluctuations are now closely spaced among each other. From left to right: $z = 0$, $z = 45$, $z = 85$, $z = 115$. In this case the formation of the 'star' in $\mathbf{k}-$space takes place before the formation of DSWs in $\mathbf{r}-$space, as revealed by the zoom of (a3) and (b3) reported in the last column (a3',b3') at $z = 85$. The random wave still evolves in the presence of a strong homogeneous (plane-wave) condensate, see the color-bar. The spectra have been plotted in $\log_{10}$-scale by removing the plane-wave condensate spectral peak at $\mathbf{k} = 0$.

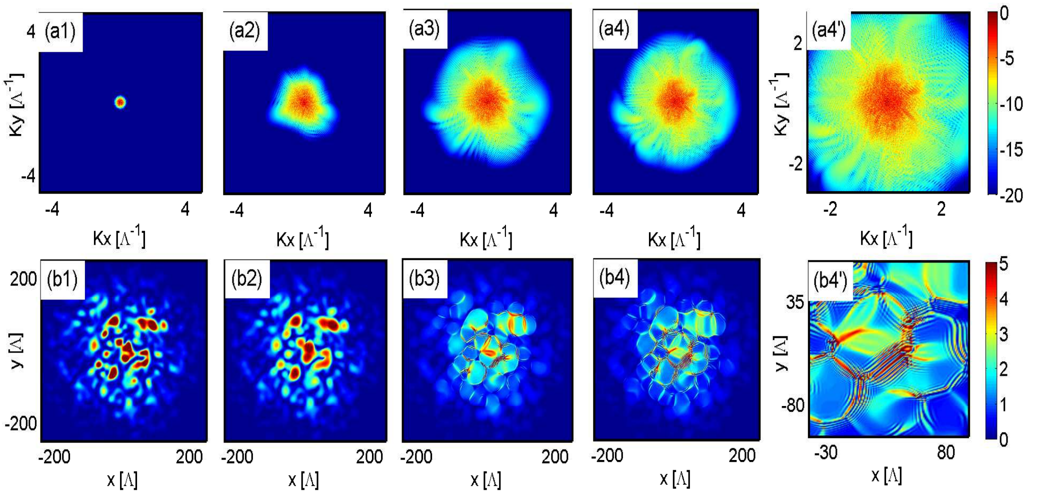

Supplementary Figure 13: **Dispersive waves.** Evolutions of the intensity and spectrum of the random wave corresponding to the numerical simulation reported in Fig. 1(b) in the main text. In this case there is no homogeneous plane-wave background, so that linear waves evolve according to the dispersion relation, $\omega(k) \propto k^2$. From left to right: $z = 0$, $z = 2$, $z = 6$, $z = 8$ – the last column (a4',b4') reports corresponding zooms of the spectrum and intensity at $z = 8$. The formation of a star in $\mathbf{k}-$space is related to the strong interaction among DSWs that emanate from different speckles and whose compression against each other leads to polygon-like patterns with effective 1D DSW sides, as revealed by the zoom in panel (b4').

The Supplemental article is organized as follows. In Note 1 we provide complementary experimental results as well as a more detailed discussion of the validity of the NLSE model equation used to describe the optical experiment. In Note 2 we provide a detailed derivation of the effective 1D long-range Vlasov equation (LRVE) used in the main paper by starting from the general 2D LRVE. In Note 3 we show that the shock singularity prevents a closure of the infinite hierarchy of moments equations and, in this way, it prevents a reduced description of the regularization of the incoherent shock. In this Note 3 we also discuss the incoherent shock phenomenon in the purely one dimensional case, $D = 1$. Finally, in Note 4, we discuss the relation between the dispersive shocklets regime and a challenging issue inherent to acoustic wave turbulence. Note that special care has been devoted to structure this Supplemental article in a self-contained fashion.

Supplementary Note 1: Comments on the experiment

A. NLSE model

As commented in the main paper, a nonlocal nonlinear response of the medium is found in several wave systems, so that the impact of nonlocality on the dynamics of coherent [1–7] or incoherent [8–11] nonlinear waves has been widely investigated through generalized nonlinear Schrödinger equations (NLSE). It should be remarked that the works reported through [8–11] refer to incoherent waves that propagate in a nonlinear material characterized by a very slow response time, a property which is known to introduce a different physics as discussed in in the review [12] or in Sec. 4 of [13]. Within this general framework, our experiment has been described theoretically by following previously elaborated NLSE models of optical wave propagation in thermal nonlinear media [14–18]

$$i\partial_z \psi + \frac{1}{2k_L}\nabla^2\psi + k_0 \Delta n\, \psi = -i\frac{\alpha}{2}\psi, \tag{1}$$

$$\Delta n - \sigma^2 \nabla^2 \Delta n = n_2 |\psi|^2, \tag{2}$$

where $\Delta n(\mathbf{r}, z)$ is the refractive index variation induced by the thermal nonlinearity, with $\Delta n = n_2|\psi|^2$ in the local limit ($\sigma = 0$), $n_2(< 0)$ being the defocusing nonlinear Kerr coefficient. The wave number $k_L = nk_0 = n2\pi/\lambda_0$, where $\lambda_0(= 0.532\mu\text{m})$ is the wavelength of the laser source and $n(= 1.364)$ the linear refractive index. The parameter α denotes the linear losses during the propagation. It is straightforward to see that the model Eqs.(1-2) can be recast in the form of NLSE (1) in the main paper, with the normalized response function

$$U(r) = \frac{K_0(r/\sigma)}{2\pi\sigma^2}, \tag{3}$$

where $K_0(x)$ denotes the modified Bessel function of second kind (zero-th order). The healing length measures the typical modulational instability period in the local nonlinear regime, it reads $\Lambda = \sqrt{L_{nl}/(2k_L)}$, where $L_{nl} = 1/(|\gamma|\rho)$ is the nonlinear length, ρ is the intensity and $\gamma = k_0 n_2$. Note that L_{nl} is defined from the intensity in the transverse surface section of the optical beam, so that it slightly differs from L_0 whose corresponding intensity is defined as the power over all the numerical window (this latter definition of L_0 proved convenient in order to obtain a direct quantitative comparison between NLSE and LRVE simulations, without using adjustable parameters). In the experimental configuration in which we reported the

observation of incoherent shock waves, we measured a variation of the refractive index of $\Delta n \simeq -2 \times 10^{-4}$ with a beam of radius 2mm and power P=5W ($\rho = 4 \times 10^5$W/m^2), so that $n_2 \simeq -5 \times 10^{-10}$m^2/W. Note that, as discussed in the main paper, in the dispersive shocklets configuration of the experiment, the concentration of graphene flakes has been increased significantly (see our discussion below), so that the measured variation of the refractive index was 2 times larger as compared to the incoherent shock experiment. Considering the above parameters, a healing length in the range $\Lambda \simeq$ 3-10μm can easily be obtained in our experiments by varying the power of the injected speckle beam.

The derivation of the model Eqs.(1-2) is based, in substance, on the approximation of the three-dimensional Fourier equation for the temperature profile by a nonlocal nonlinearity with a finite degree of nonlocality – we refer the reader to Refs.[14–19] for more details. Note in particular that this model does not describe the complex dynamics of heating-induced convection which has been shown to affect our experimental results. It should be stressed that a direct measurement of the nonlocal nonlinear length, σ, constitutes a difficult problem, see in particular Refs.[14, 18, 19] and references therein. An expression that relates the parameter σ to different parameters which characterize the nonlinear liquid was derived in [14], in which it was shown in particular that the nonlocal length scales as $\sigma \sim 1/\sqrt{\alpha}$.

In the experimental configuration in which we studied incoherent shock waves a small concentration of graphene flakes was used, while such concentration was substantially increased for the study of the formation of dispersive shocklets. The incoherent shock experiment requires a long-range interaction regime with a highly nonlocal nonlinearity, a property that has been obtained thanks to the very low absorption of the solution of graphene flakes. Indeed, in the absence of fluoresence mechanisms that dissipate a large portion of the input beam energy into emitted light (at only a slightly lower frequency), the dilute solution of graphene flakes provides optimal conversion of absorbed laser energy into heat and thus requires much lower absorption for a given temperature increase with respect to other liquids used e.g. in [14, 18]. Considering the estimation of the nonlocal length derived in Ref.[14] and the value of the absorption coefficient measured in the incoherent shock experiment ($\alpha = 0.013$cm^{-1}), we obtain a nonlocal length of $\sigma \simeq 400\mu$m. This value is typically one order of magnitude larger than typical values considered in Refs.[14, 18]. This results from the much smaller absorption of our solution of graphene flakes as compared to usual highly absorbing dye (rhodamine [14], iodine [16]). Moreover, the estimation of $\sigma \simeq 400\mu$m should be considered as a lower bound, since it was shown in Ref.[19] that for extended nonlinear samples such as those used in our incoherent shocks experiments the nonlocal length may be larger, $\sigma \sim 900$ μm. Also remark that an indirect measurement of σ based on a measurement of the dispersion relation (that depends on σ) yields a value of $\sigma \sim 400$ μm. These results will be reported elsewhere (manuscript in preparation).

In summary, although we do not have a precise value for the parameter σ, according to the previous discussion we can consider that the nonlocal length lies in the range, $400\mu\text{m} < \sigma < 900\mu\text{m}$, for the incoherent shock experiment. Then regardless of the precise value of σ (which does not itself modify the physics), we are able to guarantee a correct separation of length scales in the incoherent shock measurements, i.e., $\sigma > \lambda_c^0 \gg \Lambda$. On the other hand, in the experiment aimed at studying the formation of dispersive shocklets, the concentration of graphene flakes has been significantly increased so as to increase the absorption ($\alpha = 1.7$cm^{-1}), and thus reduce the nonlocal length. According to the scaling $\alpha \sim 1/\sqrt{\alpha}$ [14], the nonlocal length has been reduced by a factor ∼10. *This significant reduction of the nonlocal length has been confirmed by the numerical simulations of the NLSE (1-2): A value of $\sigma > 500\mu m$ was necessary in order to reproduce the incoherent shock experimental results, while accurate simulations of shocklets experimental results required a significant reduction of the nonlocal length, typically $\sigma < 80\mu m$.* Note in particular that

the values of the nonlocal length used to simulate the two experimental conditions ($\sigma \simeq 600\mu$m for the incoherent shock, and $\sigma \simeq 60\mu$m for the dispersive shocklets, see Figs. 3-4 in the main paper) verify the scaling $\sigma \sim 1/\sqrt{\alpha}$.

B. Complementary experimental results

1. Incoherent shock regime

We report in this section additional results in the experimental configuration in which we studied the incoherent shock wave phenomenon (see Fig. 4 in the main paper). We show in particular that, by decreasing the correlation length of the injected speckle, the incoherent shock phenomenon can no longer be clearly identified. This experimental observation is reported in Fig. 1, and it has been confirmed by the numerical simulations in Fig. 2. Note that, as discussed in the main paper, the asymmetry in the lower part of the experimental beam profile in Fig. 1 is due to convection within the sample (also note that the images are inverted due to the system imaging - the lower part of the figure corresponds to the upper part of the beam in the actual experiment). Convection introduces a temporally varying pattern that washes out the speckle distribution during measurements. However, convection does not modify the evolution elsewhere within the beam, as demonstrated in other regimes [16].

We can remark in Figs. 1-2 that a main impact of the reduced correlation length of the injected beam is a slowing down of the dynamics of the incoherent shock. The slowing down of the dynamics can be simply explained from a qualitative analysis of the LRVE. By decreasing λ_c^0 the corresponding spectrum $S_0(\mathbf{k}) = \int n_{\mathbf{k}}(\mathbf{r}, z=0) d\mathbf{r}$ gets larger in $\mathbf{k}-$space. Since the total power (proportional to the total volume $\mathcal{N} = \iint n_{\mathbf{k}}(\mathbf{r}, z=0) d\mathbf{k} d\mathbf{r}$) is kept fixed, this leads to a decrease of the typical amplitude of $n_{\mathbf{k}}(\mathbf{r}, z=0)$, which in turn leads to a slowing down of the dynamics. This effect can be identified in Fig. 1, where we can note a coherence enhancement in the center of the beam, while the second stage of incoherent shock formation characterized by a radial flux of quasi-particles toward the boundaries of the beam (as described by the Burgers term in Eq.(27)) slows down significantly. This analysis is confirmed by the numerical simulations reported in Fig. 2. We see in particular in this figure that, as the initial correlation length (λ_c^0) decreases, the spectrogram evolves from the peculiar $Z-$shaped distortion discussed in Fig. 4 in the main paper toward an almost regular deformation which evidences the absence of the shock singularity. It is important to note that in the case of a regular deformation of the spectrogram, the beam is characterized by the same spectral width, Δk (i.e., same correlation length λ_c), irrespective of the position y, as illustrated by the skewed rectilinear shape of the spectrogram in Fig. 2(b4). This study of the spectrogram dynamics clearly shows the suppression of the incoherent shock phenomenon.

2. Dispersive shocklets regime

In this section we report additional results in the experimental configuration in which we reported the formation of dispersive shocklets from a speckle beam, see Fig. 5 in the main paper. We remind that, to observe this dispersive shocklets regime, we had to substantially reduce the effective nonlocal length in the sample, typically by a factor ~ 10 (see Sec. above). Note that, due to such a strong absorption, the power transmitted through the sample decreases significantly, so that the length of the sample has been

reduced to 1cm. Moreover, in order to inhibit long-range collective effects, the correlation length of the input speckle beam was chosen much larger than the nonlocal length, see Fig. 5 in the main paper where $\lambda_c^0(\sim 250\mu\text{m}) \gg \sigma(\sim 60\mu\text{m})$. Here, to show the importance of this requirement, we report experimental and numerical results in which the correlation length of the input speckle has been deliberately reduced with respect to this value, see Fig. 3 for $\lambda_c^0 \sim 110\mu\text{m}$. It is interesting to compare these results with those of Fig. 5 in the main paper. We note that, as the correlation length decreases there is a strong interaction among the different speckles of the input beam. This leads to an effective global dynamics of neighbouring speckles (see the big holes of low intensity regions in Figs. 3) which prevents the development of dispersive shock waves within each individual speckle of the incoherent beam. In this way, the formation of DSWs featured by periodic patterns gradually decreases and tends to disappear for $\lambda_c^0 \ll \sigma$.

C. Spectrogram measurements

We briefly comment here the method used to record the spectrograms reported in Fig. 4 in the main paper. The measurement of the spectrograms (x, k_x) were performed using the set-up shown in Fig. 4. For a fixed laser input power, we image the beam profile at the sample output using a 4-f telescope. We then place a pinhole that has a 0.5 mm diameter in the telescope imaging plane so that we select only a very small and well-defined portion of the imaged beam. The pinhole may be translated along the x direction at a fixed $y-$position. In our experiments, the $y-$position is chosen so that by scanning in x, the pinhole passes from one edge of the beam to the other through the beam center. The transmitted light through the pinhole then passes through a cylindrical lens: at a distance f=40 cm after the lens (f is the lens focal length) we obtain the transverse k_x Fourier transform that is then recorded on a CCD camera. This is repeated for several x positions by translating the pinhole across the beam and the final (x, k_x) spectrum is built-up by adding together each of the individual measurements.

Supplementary Note 2: Reduction to an effective 1D-LRVE equation

In this section we show that if the initial condition is highly nonlinear and radially symmetric (the local spectrum depends only on $k = |\mathbf{k}|$ and $r = |\mathbf{r}|$), then the 2D LRVE can be reduced to an *effective* 1D LRVE (2) in the main paper.

A. Radial symmetry: 3D phase-space reduction

The starting point is the 2D-LRVE

$$\partial_z n_{\mathbf{k}}(\mathbf{r}) + \beta \mathbf{k} \cdot \nabla_{\mathbf{r}} n_{\mathbf{k}}(\mathbf{r}) - \nabla_{\mathbf{r}} V(\mathbf{r}) \cdot \nabla_{\mathbf{k}} n_{\mathbf{k}}(\mathbf{r}) = 0, \tag{4}$$

where $\mathbf{k}, \mathbf{r} \in \mathbb{R}^2$, so that $n_{\mathbf{k}}(\mathbf{r}, z)$ evolves in a 4-dimensional phase-space. We remind that $V(\mathbf{r}, z) = \frac{\gamma}{(2\pi)^2} \int U(|\mathbf{r}'|) N(\mathbf{r} - \mathbf{r}', z) d\mathbf{r}'$, $N(\mathbf{r}, z) = \int n_{\mathbf{k}}(\mathbf{r}, z) d\mathbf{k}$. This equation conserves the 'power', $\mathcal{N} = \frac{1}{(2\pi)^2} \int N(\mathbf{r}, z) d\mathbf{r}$, the Hamiltonian $\mathcal{H}_{Vl} = \iint \omega(k)\, n_{\mathbf{k}}(\mathbf{r}, z) d\mathbf{k} d\mathbf{r} + \frac{1}{2} \int V(\mathbf{r}, z) N(\mathbf{r}, z) d\mathbf{r}$, and $\mathcal{M} = \iint f[n] d\mathbf{k} d\mathbf{r}$ where $f[n]$ is an arbitrary functional of n. If the initial condition depends only on $k = |\mathbf{k}|$ and $r = |\mathbf{r}|$ $\big(n_{\mathbf{k}}(\mathbf{r}, z = 0) = n_k(r, z = 0)\big)$, then an inspection of the LRVE (4) reveals that the solution will also depend on the angle $\cos\theta = \mathbf{k} \cdot \mathbf{r}/(kr)$ as soon as $z > 0$, viz. $n_{\mathbf{k}}(\mathbf{r}, z) = n_k(r, \theta, z)$. In particular, if

the initial condition depends only on (k, r, θ), then for $z > 0$ it remains a function of these three variables only [11]. This means that assuming initial radial symmetry, one can gain one dimension, i.e., the LRVE evolves in a 3-dimensional phase-space (k, r, θ), instead of the four-dimensional phase-space $(\mathbf{k}, \mathbf{r})$. More precisely, by considering an initial condition $n_k(r, \theta, z = 0)$, with $r, k \in [0, \infty)$, $\theta \in [0, 2\pi)$, then the local spectrum evolves according to the following LRVE:

$$\partial_z n_k(r,\theta,z) + \beta\Big(k\cos\theta\partial_r n_k(r,\theta,z) - \frac{k}{r}\sin\theta\partial_\theta n_k(r,\theta,z)\Big)$$
$$-\partial_r V\Big(\cos\theta\partial_k n_k(r,\theta,z) - \frac{1}{k}\sin\theta\partial_\theta n_k(r,\theta,z)\Big) = 0, \quad (5)$$

where

$$V(r,z) = \frac{\gamma}{(2\pi)^2}\int \tilde{U}(r,r')N(r',z)r'dr', \quad (6)$$
$$N(r,z) = \int_0^{2\pi}\int_0^\infty n_k(r,\theta,z)kdkd\theta, \quad (7)$$
$$\tilde{U}(r,r') = \int_0^{2\pi} U\Big(\sqrt{r^2 + r'^2 - 2rr'\cos\theta}\Big)d\theta. \quad (8)$$

In the numerics the initial condition of the random field $\psi_0(\mathbf{r})$ is generated in such a way that the autocorrelation $B(\mathbf{r}, \boldsymbol{\xi}, z = 0) = \langle\psi_0(\mathbf{r} + \boldsymbol{\xi}/2)\,\psi_0^*(\mathbf{r} - \boldsymbol{\xi}/2)\rangle = I_0(r)C_0(\xi)$, so that $n_{\mathbf{k}}(\mathbf{r}, z = 0) = \int B(\mathbf{r}, \boldsymbol{\xi}, z = 0)\exp(-i\mathbf{k}\cdot\boldsymbol{\xi})d\boldsymbol{\xi}$ factorizes into the product of a function of r and a function of k: The initial local spectrum, $n(k, r, \theta, z = 0)$, is a homogeneous function of θ. *However, according to LRVE (5), the local spectrum will become inhomogeneous in θ for $z > 0$. This seems to indicate that there is no possibility to further reduce the 3D phase-space of the LRVE (5). In the following we show that such reduction is possible when the system evolves in the highly nonlinear regime of interaction.*

B. Highly nonlinear regime: 2D phase-space reduction

We now show that if the initial condition is highly nonlinear ($\mathcal{U}_0 \gg \mathcal{E}_0$), then the solution of the LRVE (5) keeps the structure $n_k(r, \theta, z) = \tilde{n}_k(r, z)\delta(\theta)$ for $z > 0$. For this purpose, we consider an initial local spectrum which is homogeneous in θ but very narrow in k, of the form

$$n_{\mathbf{k}}(\mathbf{r}, z = 0) = N_0(r)\frac{1}{k_c^2}\varphi\big(\frac{k}{k_c}\big), \quad (9)$$

where k_c is a small parameter reflecting the narrowness of the initial spectrum. $\varphi(s)$ is a bell-shaped smooth function, which is maximal at $s = 0$, of typical width ~ 1, and normalized in such a way that $\int_0^\infty \varphi(s)sds = 2\pi$. Then in the early stage of the evolution, the third term in the LRVE (4) is dominant over the second one: $\partial_z n_{\mathbf{k}}(\mathbf{r}, z) \simeq \nabla_{\mathbf{r}}V(r)\cdot\nabla_{\mathbf{k}}n_{\mathbf{k}}(\mathbf{r}, z)$, with $V(r) = \frac{\gamma}{(2\pi)^2}\int\tilde{U}(r, r')N_0(r')r'dr'$. This is a linear equation whose advection-like term leads to a solution of the form

$$n_{\mathbf{k}}(\mathbf{r}, z) = N_0(r)\frac{1}{k_c^2}\varphi\Big(\frac{|\mathbf{k} + \nabla_{\mathbf{r}}V(r)z|}{k_c}\Big). \quad (10)$$

Since $\nabla_{\mathbf{r}}V(r) = \partial_r V(r)\mathbf{r}/r$, this can be approximated (for k_c small) by

$$n_{\mathbf{k}}(\mathbf{r}, z) \simeq \frac{N_0(r)}{|\partial_r V(r)|z}\,\delta\big(k - |\partial_r V(r)|z\big)\,\delta(\theta), \quad (11)$$

when $\partial_r V(r) < 0$ – note that this latter condition is always verified whenever $N_0(r)$ is maximum at $r = 0$. This shows that a narrow homogeneous spectrum gives rise to a radially outgoing flux in the early stage of the evolution, i.e.,

$$\mathbf{p}(\mathbf{r}, z) = \frac{1}{N(\mathbf{r}, z)} \int \mathbf{k}\, n_{\mathbf{k}}(\mathbf{r}, z)\, d\mathbf{k} = p(r, z)\, \frac{\mathbf{r}}{r}. \tag{12}$$

Next, one can remark from the LRVE (5) that an initial condition of the form $n_k(r, \theta, z = 0) = \tilde{n}_k(r, z = 0)\delta(\theta)$, is preserved during the evolution, viz. $n_k(r, \theta, z) = \tilde{n}_k(r, z)\delta(\theta)$. Then the equation governing the evolution of $\tilde{n}_k(r, z)$ can be derived by making use of the identity

$$\cos\theta \partial_r \big(\tilde{n}\delta(\theta)\big) - \frac{1}{r} \sin\theta \partial_\theta \big(\tilde{n}\delta(\theta)\big) = \frac{1}{r} \partial_r \big(r\tilde{n}\big)\delta(\theta), \tag{13}$$

and a similar equation with the substitution $r \to k$. Note that (13) results from the fact that for any periodic test function $\phi(\theta)$, we have

$$\begin{aligned}
&\int \Big(\cos\theta \partial_r \big(\tilde{n}\delta(\theta)\big) - \frac{\sin\theta}{r} \partial_\theta \big(\tilde{n}\delta(\theta)\big) \Big) \phi(\theta) d\theta \\
&= \int \cos\theta \partial_r \big(\tilde{n}\big)\delta(\theta)\phi(\theta) + \frac{\tilde{n}}{r}\delta(\theta)\partial_\theta\big(\sin(\theta)\phi(\theta)\big) d\theta \\
&= \int \cos\theta \partial_r \big(\tilde{n}\big)\delta(\theta)\phi(\theta) + \frac{\tilde{n}}{r}\delta(\theta)\big(\sin(\theta)\phi'(\theta) + \cos(\theta)\phi(\theta)\big) d\theta \\
&= \partial_r \tilde{n}\phi(0) + \frac{\tilde{n}}{r}\phi(0) = \frac{1}{r}\partial_r \big(r\tilde{n}\big)\phi(0).
\end{aligned}$$

Then according to the identity (13), it results that the function $\tilde{n}_k(r, z)$ is governed by the following 1D-LRVE:

$$\partial_z \tilde{n}_k(r, z) + \frac{\beta k}{r} \partial_r \big(r\tilde{n}_k(r, z)\big) - \frac{\partial_r V}{k} \partial_k \big(k\tilde{n}_k(r, z)\big) = 0 \tag{14}$$

with

$$V(r, z) = \frac{\gamma}{(2\pi)^2} \int_0^\infty \tilde{U}(r, r') N(r', z) r' dr', \tag{15}$$

$$N(r, z) = \int_0^\infty \tilde{n}_k(r, z) k dk \tag{16}$$

Finally, Eq.(14) can be further simplified by introducing $\check{n}_k(r, z) = kr\, \tilde{n}_k(r, z)$. In this way we recover the effective 1D-LRVE (2) used in the main paper:

$$\partial_z \check{n}_k(r, z) + \beta k \partial_r \check{n}_k(r, z) - \partial_r V \partial_k \check{n}_k(r, z) = 0, \tag{17}$$

where $V(r, z) = \frac{\gamma}{(2\pi)^2} \int_0^\infty \tilde{U}(r, r')\check{N}(r', z)dr'$, with $\check{N}(r, z) = \int_0^\infty \check{n}_k(r, z)dk = rN(r, z)$.

Supplementary Note 3: Shock regularization and closure

A. Hierarchy of $k-$moment equations

In this Section we consider the problem of the regularization of the incoherent shock singularities discussed in the main paper through the reduced 'hydrodynamic' model [see Eqs.(3-4) in the main text].

As we will see, this problem is related to a long-standing mathematical problem of achieving a closure of the infinite hierarchy of equations that govern the evolutions of $k-$moments in transport kinetic theories [27–29]. Before entering into details, notice that *the closure of moment equations in wave turbulence theory is known to be justified in the weakly nonlinear regime [20–26], while the closure problem considered here refers to the strongly nonlinear regime.* In the present problem, the 'hydrodynamic' model [Eqs.(3-4) in the main text] refers to the lowest-order nonlinear closure of the hierarchy of $k-$moments equations for the LRVE. As discussed in the main text, this model describes in detail the system in the strong nonlinear regime ($\mathcal{E} \ll \mathcal{U}$), i.e., it describes the formation of the shock, up to the singular point (see Fig. 3 in the main text for $D = 2$, or Fig. 6 for $D = 1$). This model exhibits a finite 'time' singularity, so that it breaks down at the shock point. As we will see, nearby the singularity linear dispersive effects become of the same order as nonlinear effects, $\mathcal{E} \sim \mathcal{U}$. This is reflected by the fact that, at the shock point, higher-order $k-$moments of the LRVE become all of the same order of magnitude, which prevents the possibility of achieving a closure.

In the following we consider for simplicity the purely one-dimensional (1D) problem. In this way we also complete the presentation of the incoherent shock phenomenon discussed in the main paper in two-dimensions (2D). For the sake of clarity, we remind here the NLSE in 1D:

$$i\partial_z \psi = -\beta \partial_{xx}\psi + \gamma\psi \int_{-\infty}^{+\infty} U(x-x')\ |\psi|^2(t,x')\ dx', \tag{18}$$

which conserves the 'power' $\mathcal{N} = \int |\psi|^2 dx$, and the Hamiltonian $\mathcal{H} = \mathcal{E} + \mathcal{U}$, where $\mathcal{E} = \beta \int |\partial_x \psi|^2\, dx$ and $\mathcal{U} = \frac{\gamma}{2} \iint |\psi(x)|^2\, U(x-y)\, |\psi(y)|^2\, dx\, dy$ denote the linear and nonlinear contributions to the energy $\mathcal{H}$. The corresponding 1D-LRVE reads

$$\partial_z n_k(x,z) + \beta k \partial_x n_k(x,z) - \partial_x V \partial_k n_k(x,z) = 0, \tag{19}$$

where $V(x,z) = \frac{\gamma}{2\pi}\int_{-\infty}^{+\infty} U(x-x')N(x',z)dx'$, and $N(x,z) = \int_{-\infty}^{+\infty} n_k(x,z)dk$, with $k, x \in \mathbb{R}$. We recall that $\gamma > 0$ for a defocusing nonlinearity. In addition to the first moment, i.e. the momentum

$$p(x,z) = \langle k \rangle (x,z), \tag{20}$$

we define the following centered $k-$moments:

$$m_q(x,z) = N(x,z) \left\langle (k-p)^q \right\rangle, \tag{21}$$

where the average is defined as $\langle \mathcal{A} \rangle (x,t) = \int \mathcal{A} n_k(x,t)dk / \int n_k(x,t)dk$. Note that $m_1(x,z) = 0$, while $m_2(x,z)$ and $m_3(x,z)$ may be interpreted in analogy with a 'pressure' and a 'heat', respectively. Starting from the LRVE (19), we derive without approximations the hierarchy of moment equations

$$\partial_z N + \beta \partial_x (N\, p) = 0, \tag{22}$$

$$\partial_z p + \beta p \partial_x p + \partial_x V = -\frac{\beta}{N}\partial_x m_2, \tag{23}$$

$$\partial_z m_q + \beta \partial_x (p m_q + m_{q+1}) = \beta q \Big(\frac{m_{q-1}}{N} \partial_x m_2 - m_q \partial_x p \Big) \tag{24}$$

for $q \geq 2$. This hierarchy is not closed because the equation for $m_q(x,z)$ depends on the corresponding higher-order moment, $m_{q+1}(x,z)$. Note that the structure of this hierarchy of moment equations exhibit properties similar to those discussed in Refs.[27–29].

B. One-dimensional (D=1) shock and collapse singularities

The hierarchy can be truncated at different orders. At the lowest order we have the mono-kinetic singular solution considered in the main paper, $n_k(x,t) = N(x,t)\delta\big(k - K(x,t)\big)$, which immediately gives

$$p(x,z) = K(x,z), \qquad m_2(x,z) = 0. \tag{25}$$

It is important to stress that this closure of the hierarchy refers to the strong nonlinear regime, i.e., the regime in which the incoherent wave is characterized by a narrow spectrum, as expressed by the presence of the Dirac $\delta-$function in the singular solution for the local spectrum. In this way the hierarchy is truncated by neglecting the second-order moment, $m_2(x,z) = 0$, and Eqs.(22-24) recover the reduced 'hydrodynamic' model considered in the main paper. In the 1D case considered here, it reads:

$$\partial_z N + \beta \partial_x (NK) = 0, \tag{26}$$

$$\partial_z K + \beta K \partial_x K + \partial_x V = 0. \tag{27}$$

Note that this model resembles the effective 1D hydrodynamic model derived in the main paper [see Eqs.(3-4) in the main text], although in this latter model the effective potential $V(r,z)$ is not a simple convolution of the intensity with the response function. The model (26-27) shows that the shock and collapse singularities reported in the main paper for $D = 2$ also occur in the one-dimensional ($D = 1$) case. Indeed, proceeding as in the main paper, it can be shown by the method of the characteristics [30] that $\partial_x K\big(X(z), z\big)$ and $N\big(X(z), z\big)$ exhibit a finite 'time' blow-up singularity, i.e., just before $z = z_\infty$:

$$\partial_x K\big(X(z), z\big) \simeq \frac{-1}{\beta(z_\infty - z)}, \tag{28}$$

$$N\big(X(z), z\big) \simeq \frac{N(x_0, 0)}{z_\infty - z}, \tag{29}$$

where the characteristics verifies $\partial_z X(z) = \beta K\big(X(z), z\big)$, and $X(0) = x_0$.

The hydrodynamic model (26-27) has been shown to provide an accurate description of the dynamics before the shock singularity in the main paper. This is illustrated in Figs. 5-6, which report the phenomenon of incoherent shock wave in the one dimensional case, $D = 1$. We stress in particular that a quantitative agreement has been obtained between the simulations of NLSE, LRVE and hydrodynamic equations, without using adjustable parameters, as remarkably illustrated in Fig. 6. Finally note that, as discussed in the main paper, the singularities described by the hydrodynamic model are regularized by the LRVE, since the spectrogram evolves in the two-dimensional phase-space and can thus become 'multi-valued'. This can be interpreted in analogy with the computation of multivalued solutions to the forced Burgers equation derived in the semi-classical limit of the linear Schrödinger equation [31].

C. Impossibility of achieving a closure

In order to describe the regularization of the singularity, we analyze the possibility of achieving a higher-order closure of the hierarchy. We report in Fig. 7 the comparison between different terms that appear in Eqs.(22-24). For this purpose, we have solved numerically the LRVE (19), whose solution, $n_k(x,z)$, has been used to compute the different terms in Eqs.(22-24). In the example considered in Fig. 7, the incoherent shock occurs at $z_s \simeq 320 L_0$. As illustrated in Fig. 7c, the term in the rhs of Eq.(23),

$-\beta\partial_x m_2/N$, becomes of the same order as the Burgers term, $\beta p \partial_x u$, as the system approaches the shock 'time' (distance), $z_s \simeq 320 L_0$. This means that, as expected, the reduced model Eqs.(26-27) breaks down near by the shock point. More importantly, the hierarchy cannot be closed by neglecting higher-order moments. This is illustrated in Fig. 7c-g, which compare the evolutions of $p(x,z)\, m_q(x,z)$ with $m_{q+1}(x,z)$ in the lhs of Eq.(24) for different values of $q = 2, 3, 6$. The same conclusion is obtained by comparing the evolutions of $m_{q-1}(x,z)\partial_x m_2(x,z)/N(x,z)$ with $m_q(x,z)\partial_x p(x,z)$ in the rhs of Eq.(24), which are found of the same order of magnitude for different values of q. *It becomes apparent that higher-order moments become all of the same order of magnitude at the incoherent shock singularity, which prevents the possibility of achieving a closure of the hierarchy of the $k-$moment equations.*

Supplementary Note 4: Dispersive shocklets and acoustic-like turbulence

In this note we comment an interesting possible connection between the coherent shocklets regime reported in our work and a long-standing challenging issue inherent to weakly dispersive turbulent systems [32, 33]. As discussed in the main text, the development of a proper kinetic formulation of random nonlinear waves that exhibit weak acoustic-like dispersion constitutes a difficult problem [24, 26, 32, 33]. In this respect, an interesting challenging issue has been pointed out in Refs.[26, 33]: Given an initial anisotropic spectral distribution of the weakly dispersive random wave, one may wonder whether the nonlinear interactions of the next order lead to a spectral isotropic redistribution, or to condensation along specific rays in $\mathbf{k}-$space. Here we consider this issue by means of NLSE simulations in the quasi-local regime ($\sigma = 2\Lambda$), in which a large amplitude homogeneous wave (condensate) has been superimposed to the turbulent fluctuations. In this way the fluctuations evolve according to the Bogoliubov dispersion relation [25], $\omega_B(k) = \left(\beta^2 k^4/4 + \gamma\beta n_0 k^2 \tilde{U}_k\right)^{1/2}$, $\tilde{U}_k$ being the Fourier transform of $U(r)$, so that for small frequency components linear waves exhibit an acoustic-like dispersion relation, $\omega_B(k) \simeq |\mathbf{k}|$, see Fig. 8. In this Bogoliubov regime, the WT theory reveals that the interactions are essentially dominated by almost collinear three-wave resonant processes [32, 33].

In order to understand the role of shocks in the stochastic dynamics of the incoherent field, we first consider a typical initial realization of the random wave characterized by the presence of a 'stronger fluctuation' at $\mathbf{r} \simeq 0$, i.e., a fluctuation whose typical amplitude is larger than those of the surrounding fluctuations. This 'stronger speckle' develops a shock singularity well before the other surrounding small speckle fluctuations. Because of the spatial inhomogeneous character of such a strong fluctuation, there exist some natural preferential directions along which the fluctuation develops steeper fronts. As a consequence of this inhomogeneous self-steepening in the spatial domain, the corresponding spectrum of the random wave exhibits an inhomogeneous broadening in $\mathbf{k}-$space – the development of the shock front along the $x-$axis leads to a spectral broadening along k_x, see Fig. 9 ($z = 15$). In the subsequent evolution, we can remark that such an inhomogeneous spectral broadening exhibits an isotropic homogenization process in $\mathbf{k}-$space, as revealed by a comparison of $z = 15$ and $z = 25$ in Fig. 9. This effect of spectral isotropic homogenization is also observed when the the strong speckle evolves alone (i.e., in the absence of the surrounding small speckles), so that it can be simply interpreted as a consequence of the progressive steepening of the different fronts of the initial asymmetric strong speckle.

At this stage we observe an interesting phenomenon. The inhomogeneous broadening of the spectrum exhibits a kind of filamentation process that occurs along specific directions in $\mathbf{k}-$space, as illustrated in Fig. 9 at $z = 45$. This effect may probably be related to a kind of condensation of the spectrum along

specific rays in $\mathbf{k}-$space. Indeed, this effect seems to be characterized by two distinct processes. (i) We observe an elongation of the rays along their own $\mathbf{k}-$directions, a feature that can be explained by the collinear three-wave resonant interaction described by the kinetic theory in the Bogoliubov regime [33] – note in this respect that ray elongation occurs in the acoustic regime, where $\omega_B(k) \simeq k$, for $|\mathbf{k}| < 0.8$, see Fig. 8. (ii) Besides the collinear three-wave interaction, we can notice that there exists a different process featured by a transfer of energy among distinct rays in $\mathbf{k}-$space, as revealed by a detailed comparison of the spectra at $z = 15$ and $z = 45$ in Fig. 9. In the following we will term this effect 'ray-condensation' (RC) by analogy with the phenomenon suggested in the challenging issue [26] – although at this stage we don't know whether this process can be described by means of a higher order WT kinetic approach.

In order to better understand this process we have performed different numerical simulations. In particular, we have considered the same initial condition (same noise realization) as in Fig. 9, except that the strong fluctuation at $\mathbf{r} \simeq 0$ has been replaced by a fully spatial symmetric perturbation of the same typical amplitude. As a consequence, the self-steepening process of the strong perturbation in $\mathbf{r}-$space, as well as the corresponding spectral broadening process in $\mathbf{k}-$space, both occur in a homogeneous fashion (compare Figs. 9-10 at $z = 15$). Interesting to note, thanks to such a symmetric spectral broadening, the process of 'RC' can be clearly identified through the formation of a 'spectral star' in $\mathbf{k}-$space, see Fig. 10 ($z = 45$).

We stress the fact that the formation of such a 'star' in $\mathbf{k}-$space is not simply due to the formation of small shocks (shocklets) in the small speckles which surround the strong fluctuation at $\mathbf{r} = 0$. We have performed simulations starting from exactly the same initial condition (same noise realization) as in Fig. 9, except that the strong fluctuation at $\mathbf{r} = 0$ has been removed. The results are reported in Fig. 11. The steepening of the fronts that develop within the small speckles are too weak to affect the evolution of the spectrum: The spectrum at $z = 45$ does not exhibit elongations along some specific rays, i.e., it does not exhibit the formation of a 'star', which is in contrast with the corresponding spectrum obtained in Fig. 9-10 in the presence of a strong fluctuation in the initial condition at $\mathbf{r} = 0$. This reveals that *the process of 'RC' discussed above through Figs. 9-10 originates from the interaction between the strong speckle and the small speckles fluctuations.* It is also interesting to note that the formation of a 'star' still occurs in $\mathbf{k}-$space, but at a larger propagation length (see Fig. 11, $z = 85$).

Let us comment on the role of DSWs. The development of the shock singularity due to the acoustic first-order truncation ($\omega_B(k) \propto |k|$) in the main central fluctuation in Figs. 9-10 is subsequently regularized by the complete Bogoliubov dispersion relation, which thus leads to the formation of a rapidly oscillating coherent DSW structure. This effect is illustrated through zooms in different regions of the spatial intensity distribution (see Figs. 9-10). In the cases analyzed above through Figs. 9-10, the formation of the DSW takes place before the processes of 'RC' and star-like formation in $\mathbf{k}-$space, which suggests that these processes can be related to the formation and the interaction of rapidly oscillating DSWs. However, we have also considered an initial random wave characterized by closely spaced speckles fluctuations, whose typical size is still of the same order as the speckles considered in Figs. 9-11. A typical result of the simulations is reported in Fig. 12. As a consequence of the closer spatial interaction among the speckles, in this case the formation of the star in $\mathbf{k}-$space takes place before the formation of DSWs within the individual speckles of the random wave, as illustrated in Fig. 12 (note the absence of DSWs in the corresponding zoom). *This indicates that spectral star-like formation and RC effects do not necessarily require the formation of coherent regular DSW structures.*

Let us now comment the relation with the results reported in our work, in which we have considered the evolution of random waves governed by a weakly nonlocal NLSE in the absence of a strong homogeneous

(condensate) wave – the linear evolution of the field is governed by the dispersion relation, $\omega(k) \propto k^2$. We remind that, despite the presence of dispersion effects, the random wave develops a sea of small DSWs ('shocklets') because the initial condition is prepared in such a way that nonlinear effects dominate linear effects. We report in Fig. 13 the evolution of the spectrum of the field that corresponds to the numerical simulation reported in Fig. 1(b) in the main text. As already commented, in this quasi-local nonlinear regime, the evolution of the random wave is featured by the formation of a sea of DSWs. The corresponding evolution of the spectrum exhibits some properties that can be interpreted in the light of the previous discussion on spectral star-like formation. In the early stage, $z = 2$, the spectrum exhibits an inhomogeneous broadening in $\mathbf{k}-$space which is apparently due to some dominant self-steepening processes that occur among the steeper fronts of the initial spatial fluctuations. In a subsequent stage the wave-breaking shock fronts are regularized by the formation of a sea of DSWs ($z = 4$ to $z = 8$), and the spectrum exhibits some kind of filamentation that reminds the formation of a star in $\mathbf{k}-$space, as discussed above through Figs. 9-12. We should note however, that in spite of this similarity, it is difficult to discern here a transfer of energy among different rays that would lead to a phenomenon of 'RC'. In this respect, we note that the formation of a star in $\mathbf{k}-$space is related here to the strong interaction among DSWs that emanate from different speckles and that get compressed against each other, which thus leads to the formation of polygon-like patterns featured by almost 1D DSW sides. This interesting property of effective 1D-DSW patterns is apparent in the zoom reported in Fig. 13 (see also Fig. 1(b4) in the main text) – while fluctuations located on the boundaries of the beam lead to circular-shaped DSW patterns.

In summary, by considering a random wave characterized by a stronger fluctuation, we have identified an isotropic spectral homogenization process, which is subsequently followed by a process analogous to 'RC', featured by the formation of a 'star' in $\mathbf{k}-$space. The formation of such a spectral star has been identified in various different circumstances as a rather robust phenomenon, although its emergence has been shown to depend on the local properties of the initial random wave fluctuations – the formation of the spectral star can be mediated by the interaction of DSWs, but it can also occur before the process of wave breaking regularization. In this respect, caution should exercised when drawing conclusions as regard the interpretation of the mechanisms underlying these spectral dynamics of the random wave. While the purely collinear resonant three-wave interaction within each individual ray should lead to the development of wave-breaking fronts, the mechanism responsible for the transfer of energy among distinct rays that leads to 'RC' remains quite obscure. In addition, a process analogous to 'RC' has not been clearly identified in the absence of a strong homogeneous plane-wave background. Actually, in the presence of linear wave dispersion ($\omega(k) \propto k^2$), the spectral filamentation process that leads to a 'star-shaped' $\mathbf{k}-$spectrum is apparently due to the formation of DSWs, whose interaction and compression against each other leads to polygon-like patterns featured by effective 1D-DSW sides. It is also important to note that the formation of such 'spectral stars' in $\mathbf{k}-$space tends to be averaged out when the number of shocks in the numerical window increases in a substantial way, simply because of the averaging of the different shock directions in $\mathbf{r}-$space. To conclude, these preliminary simulations open several interesting questions, which we will consider in a future work specifically devoted to this important issue of weakly dispersive wave turbulence.

[1] S. Turitsyn, "Spatial dispersion of nonlinearity and stability of multidimensional solitons," Theoret. and Math. Phys. **64**, 226 (1985).

[2] O. Bang, W. Krolikowski, J. Wyller, & J.J. Rasmussen, “Collapse arrest and soliton stabilization in nonlocal nonlinear media,” Phys. Rev. E **66**, 046619 (2002).

[3] A. Dreischuh, D.N. Neshev, D.E. Petersen, O. Bang, & W. Krolikowski, “Observation of attraction between dark solitons,” Phys. Rev. Lett. **96**, 043901 (2006).

[4] D. Buccoliero, A.S. Desyatnikov, W. Krolikowski, & Y.S. Kivshar “Laguerre and Hermite Soliton Clusters in Nonlocal Nonlinear Media,” Phys. Rev. Lett. **98**, 053901 (2007).

[5] B. K. Esbensen, A. Wlotzka, M. Bache, O. Bang, & W. Krolikowski “Modulational instability and solitons in nonlocal media with competing nonlinearities,” Phys. Rev. A **84**, 053854 (2011).

[6] B. K. Esbensen, M. Bache, O. Bang, & W. Krolikowski “Anomalous interaction of nonlocal solitons in media with competing nonlinearities,” Phys. Rev. A **86**, 033838 (2012).

[7] Q. Kong, M. Shen, Z. Chen, Q. Wang, R.-K. Lee, & W. Krolikowski “Dark solitons in nonlocal media with competing nonlinearities,” Phys. Rev. A **87**, 063832 (2013).

[8] W. Krolikowski, O. Bang, & J. Wyller, “Nonlocal incoherent solitons,” Phys. Rev. E **70**, 036617 (2004).

[9] K.G. Makris, H. Sarkissian, D.N. Christodoulides, & G. Assanto, “Nonlocal incoherent spatial solitons in liquid crystals,” J. Opt. Soc. Am. B **22**, 1371 (2005).

[10] C. Rotschild, T. Schwartz, O. Cohen, & M. Segev, “Incoherent spatial solitons in effectively instantaneous nonlinear media,” Nature Photonics **2** 371 (2008).

[11] J. Garnier, J.-P. Ayanides, & O. Morice, “Propagation of partially coherent light with the Maxwell-Debye equation,” J. Opt. Soc. Am. B **20**, 1409 (2003).

[12] M. Segev, & D.N. Christodoulides, “Incoherent Solitons,” in *Spatial Solitons,* edited by S. Trillo and W. Torruellas (Springer, Berlin, 2001).

[13] A. Picozzi, J. Garnier, T. Hansson, P. Suret, S. Randoux, G. Millot, & D. Christodoulides, “Optical wave turbulence: Toward a unified nonequilibrium thermodynamic formulation of statistical nonlinear optics,” Physics Reports **542**, 1-132 (2014).

[14] N. Ghofraniha, C. Conti, G. Ruocco, & S. Trillo, “Shocks in Nonlocal Media,” Phys. Rev. Lett. **99**, 043903 (2007).

[15] A.I. Yakimenko, Y. A. Zaliznyak, & Y.S. Kivshar, “Stable vortex solitons in nonlocal self-focusing nonlinear media,” Phys. Rev. E **71**, 065603(R) (2005).

[16] C. Barsi, W. Wan, C. Sun, & J.W. Fleischer, “Dispersive shock waves with nonlocal nonlinearity,” Opt. Lett. **32**, 2930 (2007).

[17] C. Conti, A. Fratalocchi, M. Peccianti, G. Ruocco, & S. Trillo, “Observation of a Gradient Catastrophe Generating Solitons,” Phys. Rev. Lett. **102**, 083902 (2009).

[18] N. Ghofraniha, L. Santamaria Amato, V. Folli, S. Trillo, E. Del Re, & C. Conti, "Measurement of scaling laws for shock waves in thermal nonlocal media," Opt. Lett. **37**, 2325 (2012).

[19] A. Minovich, D.N. Neshev, A. Dreischuh, W. Krolikowski, & Y.S. Kivshar, "Experimental reconstruction of nonlocal response of thermal nonlinear optical media," Opt. Lett. **32**, 1599 (2007).

[20] D.J. Benney, & P.G. Saffman, "Nonlinear interactions of random waves in a dispersive medium," Proc. R. Soc. Lond. A **289**, 301-320 (1966).

[21] D.J. Benney, & A.C. Newell, "Sequential time closures of interacting random waves," J. Math. Phys. **46**, 363-393 (1967).

[22] D.J. Benney, & A.C. Newell, "Random wave closures," Stud. Appl. Math. **48**, 29-53 (1969).

[23] A.C. Newell, S. Nazarenko, & L. Biven, "Wave turbulence and intermittency," Physica D **152-153**, 520-550 (2001).

[24] V.E. Zakharov, V.S. Lvov, & G. Falkovich, *Kolmogorov Spectra of Turbulence I* (Springer, Berlin, 1992).

[25] S. Nazarenko, *Wave Turbulence* (Springer, Lectures Notes in Physics, 2011).

[26] A.C. Newell, & B. Rumpf, "Wave Turbulence," Annu. Rev. Fluid Mech. **43**, 59-78 (2011).

[27] See, e.g., C.D. Levermore, "Moment Closure Hierarchies for Kinetic Theories," J. Stat. Phys. **83**, 1021 (1996).

[28] J. Gibbons, D.D. Holm, & C. Tronci, "Vlasov moments, integrable systems and singular solutions," Phys. Lett. A **372**, 1024 (2008).

[29] M. Perin, C. Chandre, P.J. Morrison, & E. Tassi, "Higher order Hamiltonian fluid reduction of Vlasov equation," Annals of Phys. **348**, 50 (2014), and references therein.

[30] L.C. Evans, *Partial Differential Equations*, AMS, Providence, 2002, Ch. 3, Sec. 2.

[31] Shi Jin, & Xiantao Li, "Multi-phase computations of the semiclassical limit of the Schrödinger equation and related problems: Whitham vs Wigner," Physica D **182**, 46 (2003).

[32] A.C. Newell, & P.J. Aucoin, "Semidispersive wave systems," J. Fluid Mech. **49**, 593-609 (1971).

[33] V.S. L'vov, Yu. L'vov, A.C. Newell, & V. Zakharov, "Statistical description of acoustic turbulence," Phys. Rev. E **56**, 390-405 (1997).
